# Influenza A virus evolution and spatio-temporal dynamics in Eurasian wild birds: a phylogenetic and phylogeographical study of whole-genome sequence data

**DOI:** 10.1099/vir.0.000155

**Published:** 2015-08

**Authors:** Nicola S. Lewis, Josanne H. Verhagen, Zurab Javakhishvili, Colin A. Russell, Pascal Lexmond, Kim B. Westgeest, Theo M. Bestebroer, Rebecca A. Halpin, Xudong Lin, Amy Ransier, Nadia B. Fedorova, Timothy B. Stockwell, Neus Latorre-Margalef, Björn Olsen, Gavin Smith, Justin Bahl, David E. Wentworth, Jonas Waldenström, Ron A. M. Fouchier, Miranda de Graaf

**Affiliations:** ^1^​ Department of Zoology, University of Cambridge, Downing Street, Cambridge CB2 3EJ, UK; ^2^​ Department of Viroscience, Erasmus MC, PO Box 2040, 3000 CA, Rotterdam, The Netherlands; ^3^​ Institute of Ecology, Ilia State University, 3/5 Cholokashvili, Tbilisi, Georgia; ^4^​ Department of Veterinary Medicine, University of Cambridge, Madingley Road, Cambridge CB3 0ES, UK; ^5^​ J. Craig Venter Institute, Rockville, MD, 20850, USA; ^6^​ Centre for Ecology and Evolution in Microbial Model Systems, Linnaeus University, Kalmar, Sweden; ^7^​ Department of Population Health, College of Veterinary Medicine, Southeastern Cooperative Wildlife Disease Study, University of Georgia, Athens, GA, 30602, USA; ^8^​ Department of Medical Sciences, Zoonosis Science Center, Uppsala University, Uppsala, Sweden; ^9^​ Laboratory of Virus Evolution, Program in Emerging Infectious Diseases, Duke-NUS Graduate Medical School, Singapore; ^10^​ Center for Infectious Diseases, The University of Texas School of Public Health, Houston, TX, 77030, USA

## Abstract

Low pathogenic avian influenza A viruses (IAVs) have a natural host reservoir in wild waterbirds and the potential to spread to other host species. Here, we investigated the evolutionary, spatial and temporal dynamics of avian IAVs in Eurasian wild birds. We used whole-genome sequences collected as part of an intensive long-term Eurasian wild bird surveillance study, and combined this genetic data with temporal and spatial information to explore the virus evolutionary dynamics. Frequent reassortment and co-circulating lineages were observed for all eight genomic RNA segments over time. There was no apparent species-specific effect on the diversity of the avian IAVs. There was a spatial and temporal relationship between the Eurasian sequences and significant viral migration of avian IAVs from West Eurasia towards Central Eurasia. The observed viral migration patterns differed between segments. Furthermore, we discuss the challenges faced when analysing these surveillance and sequence data, and the caveats to be borne in mind when drawing conclusions from the apparent results of such analyses.

## Introduction

Low pathogenic avian influenza (LPAI) viruses have been isolated from more than 136 species of wild birds, most commonly from ducks, but also from other Anseriformes (geese and swans) and Charadriiformes (mainly gulls, waders and terns) (Alexander, 2000; Olsen *et al.*, 2006; Webster *et al.*, 1992). These bird groups have diverse annual life cycles and many are highly migratory, thereby potentially affecting spatial and temporal dynamics of avian influenza virus (AIV) at different geographical scales. Many species also frequent habitats where there is potential for direct or indirect contact with domestic birds (Webster *et al.*, 1992), primarily ducks and geese, with the concurrent risk of cross-species transmission of AIVs into domestic animals. This incursion of virus from the wild bird reservoir may have several animal and human health implications, including the risk of emergence of highly pathogenic avian influenza (HPAI) viruses and threat to food security. It also provides a means by which AIV might be brought into closer proximity to humans (Newman *et al.*, 2012). For Eurasia, waterbird migration can be broadly divided in five flyways: East Atlantic flyway, Black Sea–Mediterranean flyway, East Africa–West Asia flyway, Central Asia flyway and the East Asia Australian flyway. It should be noted that these flyways are oversimplifications and numerous exceptions exist (Hoyo *et al.*, 2011; Munster *et al.*, 2007; Olsen *et al.*, 2006; van de Kam *et al.*, 2004). Bird migration along the Central Asian flyway was reported to correlate with outbreaks of HPAI H5 and emphasized the need for bird surveillance (Newman *et al.*, 2012). Despite widespread surveillance (Olson *et al.*, 2014), there remain substantial unanswered questions about the spatial, temporal and ecological role of the host populations in defining the genetic structure of AIVs, and in inferring the role wild birds might play in trans-locating AIV from one geographical region to another. Such information is key for considering measures to reduce the risk of pathogen emergence from wildlife host reservoirs.

Previous work on identifying predictors of HPAI virus H5N1 occurrence have shown that human population size, duck density, rice cropping intensity, wild bird migration and poultry trade all contribute to virus prevalence and potential for detection (Takekawa *et al.*, 2010, 2013). Ideally, we would also want to use such spatial risk map approaches to better understand the ecology of LPAI viruses in wild birds, prior to any transmission to domestic birds. The challenges to such analyses are large as there are numerous host species with different ecological dynamics covering broad and far-reaching areas in short time frames and differences in intrinsic reservoir capacities. The prevalence of AIVs in their natural hosts depends on geographical location, seasonality, immune processes and species (Munster *et al.*, 2007; Olsen *et al.*, 2006; Vijaykrishna *et al.*, 2013). The ecological drivers of these prevalence fluctuations and how they affect viral genetic diversity are less well-characterized (Fouchier & Munster, 2009; Latorre-Margalef *et al.*, 2014; van Dijk *et al.*, 2014). Previous studies to investigate patterns in the genetic diversity among wild bird AIVs have focused predominantly on North America, partly because of the existence of larger longitudinal AIV surveillance datasets in wild birds. Studies on North American wild birds documented a high rate of genome reassortment (Dugan *et al.*, 2008), and a significant viral clustering by time and location of sampling (Chen & Holmes, 2009). Other work suggested that ducks in Alberta were representative of the total AIV diversity in North American Anseriformes and, whilst there might be spatial segregation to a particular migratory flyway over short time frames, the long-term persistence of AIV was independent of bird flyways with migration between populations throughout North America (Bahl *et al.*, 2013). Extensive surveillance studies of AIV in ducks and shorebirds in North America have permitted analyses of reassortment rates, selection pressures and patterns of genetic diversity, but until recently there has only been limited whole-genome sequence data available for AIVs in Eurasia, Africa, South America and Oceania. AIVs found in Eurasian wild birds are predominantly genetically distinct from those of wild birds in the Americas (Dugan *et al.*, 2008; Krauss *et al.*, 2004; Obenauer *et al.*, 2006), representing major geographical/continental lineages. Wild bird migratory flyways are different in Eurasia; thus patterns characterized for the Americas could differ substantially from those in Eurasia.

To explore the evolutionary and ecological dynamics of AIV in Eurasian wild birds, we used whole-genome sequences of AIVs isolated from several Anseriformes species sampled in West Eurasia along the East Atlantic flyway as part of an intensive wild bird surveillance study. These full-genome sequences were combined with genetic data of AIVs isolated throughout Eurasia. This large-scale study describes gene reassortment and viral migration within Eurasia in the light of wild bird migration and supports new directions in wild bird AIV surveillance.

## Results and Discussion

To study the spatio-temporal dynamics of AIVs in wild birds in Eurasia, more than 100 virus isolates collected from 1999 to 2007 were selected for full genomic sequencing of the coding regions. These virus isolates represented a diverse range of wild bird hosts, and included different subtypes and sampling locations predominantly within West Eurasia (Table 1). In addition, AIV full-genome sequences spanning NA1–NA9 and HA1–HA12 were retrieved from GenBank (Table S1, available in the online Supplementary Material). To focus on evolution of LPAI viruses in wild birds, we excluded all sequences from domestic birds and all sequences related to poultry outbreaks, particularly HPAI H5N1, H7 and H9.

**Table 1.** Number (*n*) of sequences per host species, country, year of isolation and subtype (*N* = 211 complete genomes)

Species	*n* ^*^	Species category	Country	*n* ^*^	Year	*n* ^*^	Subtype	*n* ^*^
Mallard	75 (57)	Dabbling duck	Netherlands	52 (51)	1956	2	H3N8	24 (4)
Duck	55	Dabbling duck	Australia	34	1963	1	H5N2	15 (4)
Red-necked stint	12	Shorebird	Sweden	32 (32)	1972	1	H4N6	14 (4)
Black duck	5	Dabbling duck	China	12	1973	1	H5N3	12 (1)
Common teal	5 (4)	Dabbling duck	Hong Kong	12	1975	3	H11N9	9 (5)
Gadwall	5 (1)	Dabbling duck	Russia	11	1976	1	H4N8	9 (1)
Gray teal	4	Dabbling duck	Italy	9	1977	3	H1N1	8 (1)
Eurasian wigeon	3 (3)	Dabbling duck	France	8	1978	9	H6N1	8 (5)
Northern shoveller	3 (2)	Dabbling duck	Japan	8	1979	8	H6N2	8 (5)
Shearwater	3	Shorebird	Mongolia	7	1980	7	H9N2	8 (4)
Teal	3	Dabbling duck	Germany	6	1981	1	H7N7	7 (6)
Bar-headed goose	2	Geese	Denmark	3	1982	1	H10N4	5 (2)
Bewick's swan	2 (2)	Swan	Taiwan	3	1983	4	H4N2	5 (2)
Black-headed gull	2 (2)	Shorebird	UK	3	1984	3	H5N1	5
Common eider	2 (2)	Diving and other ducks	Czech Republic	2	1985	2	H7N1	5 (1)
Goose	2	Geese	New Zealand	2	1986	1	H8N4	5 (4)
Northern pintail	2 (1)	Dabbling duck	Portugal	2	1988	1	H12N3	4
Ruddy shelduck	2	Diving and other ducks	Belgium	1	1991	1	H2N2	4 (2)
Sharp-tailed sandpiper	2	Shorebird	Malaysia	1	1992	1	H2N9	4 (1)
White-fronted goose	2 (2)	Geese	Slovenia	1	1992	1	H6N5	4 (1)
Dunlin	1 (1)	Shorebird	Spain	1	1994	1	H11N2	3 (2)
Eurasian coot	1	Shorebird	Ukraine	1	1998	1	H2N3	3 (3)
Fowl	1	Fowl			1999	12 (9)	H3N2	3 (1)
Garganey	1	Dabbling duck			2000	7 (4)	H3N6	3 (1)
Greylag goose	1 (1)	Geese			2001	5 (2)	H10N7	2 (2)
Gull	1	Shorebird			2002	21 (18)	H10N9	2 (1)
Herring gull	1 (1)	Shorebird			2003	10 (7)	H11N8	2 (2)
Mute swan	1 (1)	Swan			2004	14 (1)	H12N9	2
Pink-footed goose	1 (1)	Geese			2005	29 (15)	H4N3	2 (2)
Red-crested pochard	1	Diving and other ducks			2006	30 (18)	H6N8	2 (2)
Slaty-backed gull	1	Shorebird			2007	19 (9)	H7N2	2 (1)
Spot-billed duck	1	Dabbling duck			2008	6	H7N3	2 (1)
Swan	1	Swan			2009	4	H7N9	2 (1)
Tufted duck	1	Diving and other ducks					H10N1	1 (1)
Ruddy turnstone	1 (1)	Shorebird					H10N8	1 (1)
Wedge-tailed shearwater	1	Shorebird					H11N1	1 (1)
Whooper swan	1	Swan					H11N6	1
Barnacle goose	1 (1)	Geese					H1N4	1 (1)
Tern	1	Dabbling duck					H1N5	1 (1)
Whistling swan	1	Swan					H3N1	1 (1)
							H3N5	1 (1)
							H4N4	1
							H4N5	1 (1)
							H5N6	1
							H5N7	1
							H5N9	1 (1)
							H6N9	1
							H7N6	1
							H7N8	1 (1)
							H9N6	1
							H10N6	1 (1)

* The number of newly submitted sequences is given within parentheses.

**Fig. 1.**
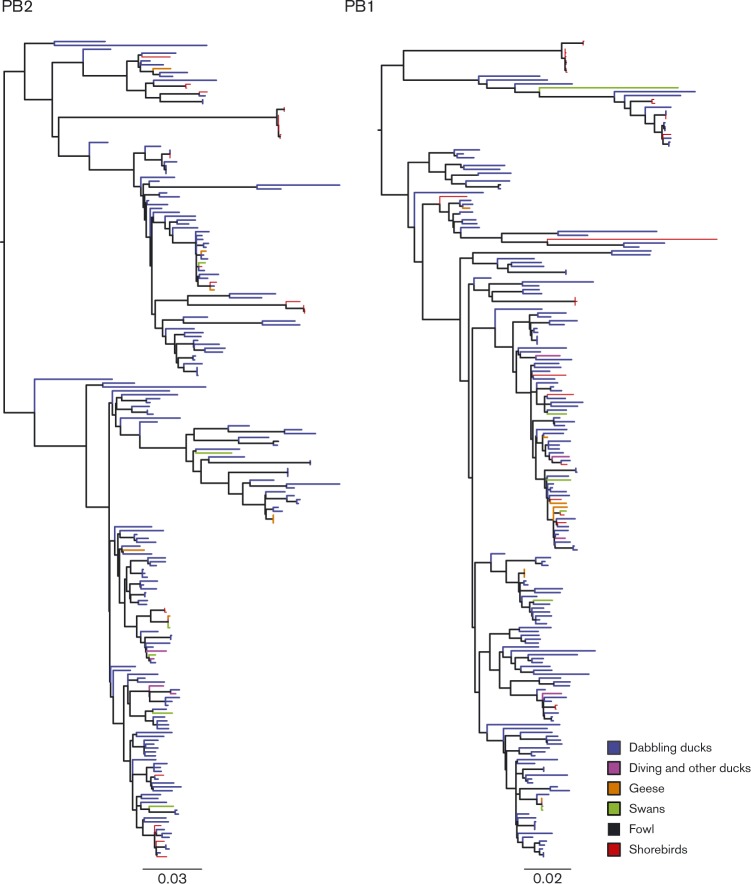
ML trees for PB2 and PB1 displaying the genetic diversity of avian IAVs in Eurasian wild birds. The taxa colour indicates the bird species group from which the sample was isolated.

Although AIVs have been isolated from more than 136 species of birds, the role of each of these species in maintaining virus diversity and virus spread is unclear. Differences in AIV prevalence and in prevalence of haemagglutinin (HA) subtypes and HA/neuraminidase (NA) subtype combinations have been observed among wild bird species (Latorre-Margalef *et al.*, 2014; Munster *et al.*, 2007). However, it is possible that for specific host species certain AIV subtypes are endemic, allowing for genetic evolution and diversification of the virus, whereas in other host species this AIV subtype is more likely to be a transient pathogen and does not become established. Here, the role of host species on influenza virus diversity was investigated using maximum-likelihood (ML) trees coloured by the bird species group from which the virus was sampled (Fig. 1; also see Fig. S1 for ML trees of all segments with strain names and Table 1 for host categories). Overall, no clear species-specific patterns could be identified. The observed genetic diversity did not seem to originate from a particular host nor were there genetic lineages limited to a single species. Nevertheless, our sequence dataset was biased with respect to bird species as the majority of AIVs included in our study were isolated from dabbling ducks (Table 1). Dabbling ducks more frequently harbour AIVs and therefore they are a ‘target species group’ for surveillance (Olsen *et al.*, 2006). Due to the over-representation of dabbling ducks, we cannot exclude that the lack of species-specific patterns in the tree topology is an artefact. Most of the Eurasian shorebird sequences appeared to cluster together in the ML tree of the HA gene, suggesting a species-specific niche wider than the H13 and H16 niche, which has been reported previously for gulls in Eurasia and North America (Hinshaw *et al.*, 1982; Krauss *et al.*, 2004; Lewis *et al.*, 2013) (Fig. S1). It should be noted that in our dataset most shorebird sequences were sampled in Oceania and were much older compared with the other Eurasian AIV sequences. Thus, our findings suggest that there is no strong species effect associated with virus diversity, similar to the results described previously for North American AIV (Chen & Holmes, 2009).

**Fig. 2.**
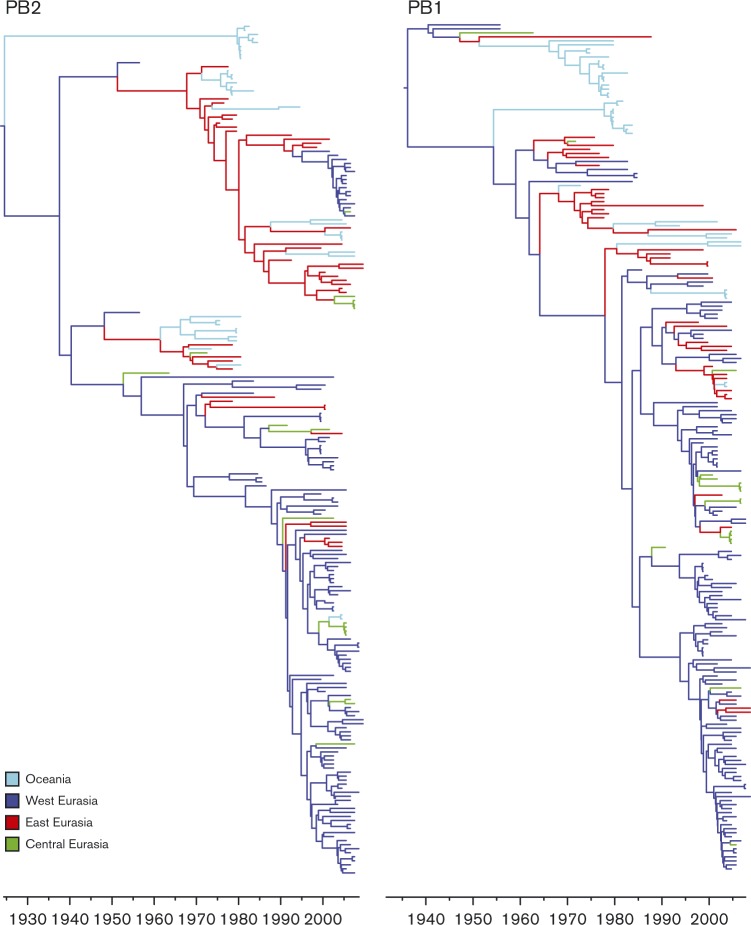
Maximum clade credibility (MCC) trees summarizing the results of the Bayesian phylogenetic inference of PB1 and PB2, and displaying the genetic diversity in different locations in Eurasia. The taxa colour indicates the regional location from where the sample was isolated. Year is indicated.

To investigate how the genetic diversity partitioned according to geographical location, ML trees were coloured by four discrete regions; West (i.e. West Eurasia), East (i.e. East Eurasia), Central (i.e. Central Eurasia) and Oceania (Fig. S2). These four geographical regions also approximate migratory flyways: West Eurasia lies within the East Atlantic flyway, Central Eurasia lies within the Black Sea–Mediterranean and Central Asian flyway, and East Eurasia and Oceania represent the East Asian–Australasian flyways. Despite overlap in migratory flyways among these four regions, viruses sampled from one geographical region and from a particular time period were most closely related to other viruses sampled from the same geographical region and could be related to different migration patterns. To further investigate the spatial and temporal processes, beast was used to infer Bayesian phylogenetic trees in which all viruses were assigned to the four discrete regions (Figs. 2 and S3).

The Bayesian analysis revealed that for all internal segments, except for NS, the most recent common ancestors (MRCAs) containing these segments circulated ∼72–108 years ago (Table 2, Fig. 2; see Fig. S3 for Bayesian trees of all segments with strain names). This recent ancestry is suggestive of hemispheric sweeps of all genetic diversity in fairly recent times, as suggested previously by others (Worobey *et al.*, 2014). The genetic diversity for the HA, NA and NS gene segments was maintained, corresponding with MRCAs much older than those of the other gene segments (PB2, PB1, PA, NP and M). However, the genetic diversity within each HA and NA subtype and NS allele was similar to that of the internal segments. For HA and NA, it was proposed that immunity in previously exposed bird populations allows the maintenance of multiple subtypes (Worobey *et al.*, 2014). It has also been described that NS alleles A and B differentially suppress innate immune responses (Munir *et al.*, 2011), perhaps allowing for maintenance of both alleles. Despite generally short times to the MRCA for the internal segments, multiple lineages co-circulated within the same years at the same locations. In our dataset, there was a high sample density of West Eurasian AIVs isolated between 2002 and 2009. However, despite this high sampling density, the genetic diversity found in West Eurasia did not completely represent the genetic diversity of AIVs throughout Eurasia during that time period. For example, for PB1 there is a lineage containing AIVs isolated from East and Central Eurasia and Oceania of which the common ancestor to the most closely related AIV from West Eurasia circulated >20 years ago. Despite probable host population and ecological differences between Eurasia and North America (Munster *et al.*, 2007), we found similar nucleotide substitution rates for Eurasian AIV strains compared with previous studies including both North American and Eurasian AIV sequences (Chen & Holmes, 2010).

**Table 2.** Rates of nucleotide substitution and times to the MRCA

Gene segment	Mean nucleotide substitution rate (10^− 3^ substitutions per site per year)	Time to MRCA (95 % higher posterior density interval) (years)
PB2	2.06 (1.80–2.32)	85 (66–111)
PB1	2.18 (1.94–2.44)	73 (64–81)
PA	1.99 (1.74–2.25)	78 (69–87)
HA	2.39 (1.91–2.88)	1003 (696–1340)
NP	1.78 (1.50–2.05)	109 (76–146)
NA	2.51 (1.99–3.08)	1294 (906–1673)
MP	1.29 (1.01–1.59)	92 (62–140)
NS	2.43 (1.70–3.18)	271 (147–428)

To test if more closely related viruses were more likely to share the same location than expected by chance alone (Parker *et al.*, 2008), Bayesian trees were analysed for evidence of taxa association by location of sampling using the Bayesian Tip-significance (BaTS) package. When the entire period of sampling was analysed, we found strong clustering by location for all regions and all gene segments (data not shown). Although this indicated that there was a spatial component to the dataset and regional maintenance of a particular clade, it could also have been due to a bias in sampling during a particular time period. To reduce this bias, the same analysis was performed using time periods of 5 years (Table 3). Despite the shorter time period, there was significant clustering of viruses isolated from the same location. Ideally, 1 year would be most relevant to the annual life cycle – and thus annual migration – of the host; this dataset comprised insufficient data for statistical power to analyse just 1 year. This illustrates one of the confounders with these data. When one attempts to reduce potential sampling bias or inconsistent sampling effort throughout the region, and capture diversity on a timescale that is relevant to the host species, one likely reduces statistical power. See Table 1 for further details on sampling by species, time and sampling site.

**Table 3.** Support for geographical clustering, based on BaTS testing (*P* values)

		Gene segment							
Location	Period	PB2	PB1	PA	HA	NP	NA	MP	NS
Central Eurasia	2001–2005	*0.01*	*0.01*	*0.01*	*0.01*	*0.01*	*0.01*	*0.01*	*0.01*
Central Eurasia	2006–2010	*0.01*	*0.01*	0.06	*0.01*	*0.01*	*0.01*	*0.01*	*0.01*
East Eurasia	1976–1980	0.11	*0.01*	*0.01*	0.11	*0.01*	*0.01*	*0.01*	*0.02*
East Eurasia	1996–2000	0.06	*0.03*	*0.02*	*0.03*	*0.02*	*0.04*	*0.02*	*0.01*
East Eurasia	2001–2005	*0.05*	*0.01*	0.15	0.14	*0.01*	0.06	*0.01*	0.10
East Eurasia	2006–2010	*0.03*	*0.01*	1.00	*0.02*	0.06	1.00	1.00	1.00
Oceania	1971–1975	*0.02*	*0.02*	*0.02*	1.00	*0.01*	1.00	*0.01*	*0.01*
Oceania	1976–1980	*0.01*	*0.01*	*0.01*	*0.01*	*0.02*	*0.01*	*0.01*	*0.01*
Oceania	1981–1985	*0.01*	*0.01*	*0.01*	*0.02*	0.06	0.06	*0.02*	*0.01*
Oceania	2001–2005	*0.05*	*0.05*	*0.03*	*0.02*	*0.04*	*0.04*	*0.04*	0.06
West Eurasia	1981–1985	*0.01*	*0.01*	*0.05*	*0.01*	*0.05*	*0.02*	1.00	*0.01*
West Eurasia	1996–2000	*0.01*	*0.01*	*0.02*	*0.01*	*0.01*	*0.02*	*0.01*	*0.01*
West Eurasia	2001–2005	*0.02*	*0.01*	0.28	0.09	*0.01*	0.11	*0.01*	*0.01*

Significant clustering of sequences from the four geographical regions was investigated by coding the regional location from which the virus was sampled onto the tips (taxa) of 900 posterior sampled trees, generating 100 null distributions, and testing the significance of the observed data. *P* ≤ 0.05 indicates significant geographical clustering, whilst *P*>0.05 indicates that traits were randomly distributed across the phylogeny. Significant values are given in italics. Only datasets with at least three sequences were included.

**Fig. 3.**
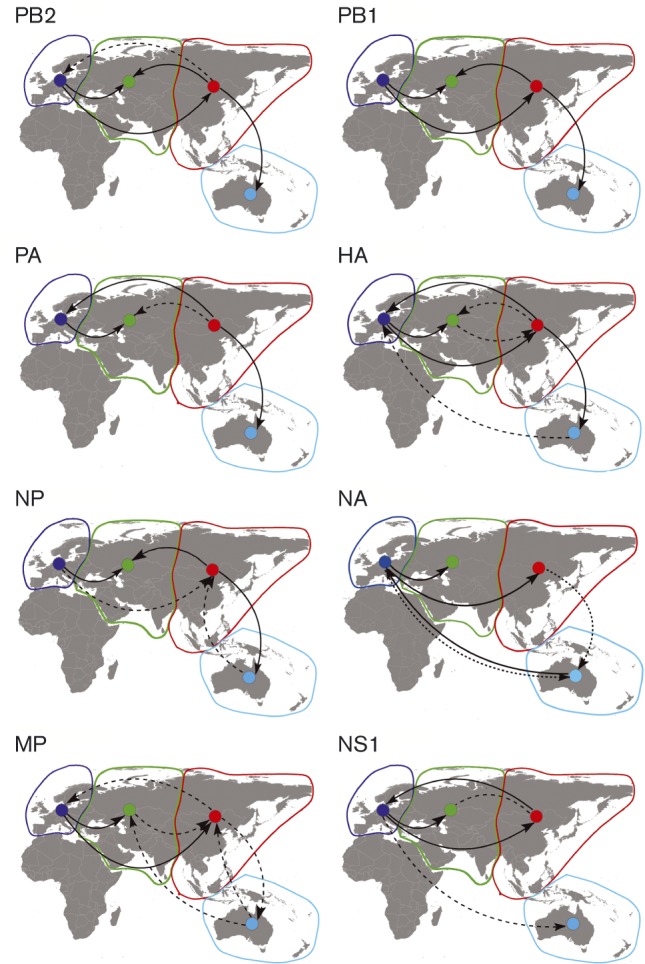
Patterns of viral migration among regions, visualized for each segment on a Eurasian map. A Bayes factor (BF) test was applied to assess the statistical support for viral migration between the discrete geographical states. Lines connecting discrete regions (cyan, Oceania; red, East Eurasia; green, Central Eurasia; blue, West Eurasia) indicate statistically supported ancestral state changes. Dotted lines, 8 ≤ BF ≤ 100 (supported); solid lines, BF>100 (strongly supported).

The Bayesian analysis was also used for ancestral state reconstruction of geographical location and to estimate the rate of viral migration among the geographical regions (Fig. 3, Table S2) (Lemey *et al.*, 2009). The highest rates of viral migration were observed from Eastern Eurasia to Oceania for PB2, Western to Eastern Eurasia for PB1, HA, NP, NA and M, and Eastern to Western Eurasia for PA and NS. Such a lack of consistent and directional spatial pattern among gene segments was also observed for North American strains (Bahl *et al.*, 2013). The inconsistent directionality observed here was likely due to differences in sampling bias and the high rates of reassortment. For HA and NA, the different subtypes are highly divergent and ancestral state reconstruction will also include modelling on the long inter-subtype branches potentially influencing the results. It should also be noted that due to sparse sampling before 1999, migration events inferred for older viruses were much more susceptible to sample bias.

**Fig. 4.**
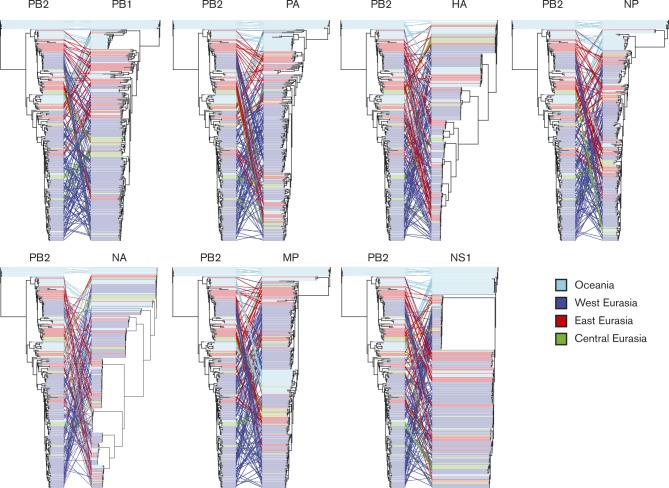
Tanglegrams constructed by rooting the MCC nucleotide substitution trees by older Australian strains. Corresponding taxa in the two trees are connected by a line. In the absence of reassortment one would expect to see a horizontal, or near horizontal, line connecting taxa between trees. The connecting lines are coloured by the region of sampling of the taxa. We show only the tree comparison for each segment with PB2 as the reference topology, but reassortment patterns were similar when other gene segments were used as the reference.

We assessed reassortment by rooting the Bayesian maximum clade credibility (MCC) nucleotide substitution trees by older Australian strains and making tanglegrams (Fig. 4). Tanglegrams enable visualization of the locations of particular taxa within the PB2 tree and each of the trees of the other segments. In the absence of reassortment, the taxa should have a nearly horizontal linkage. The tanglegram patterns indicate that there was extensive reassortment, but without completely distorting clustering between sequences of the same geographical region. Viruses of a particular subtype do not necessarily have the same genetic makeup, even for a particular species, location or year. For NS, we observed co-circulation of the A and B alleles, and similar to HA and NA, these two alleles were not associated with separate lineages for other segments. Differences in reassortment rates between the internal segments of AIVs belonging to different subtypes have been reported for Eurasian AIVs (Lu *et al.*, 2014). In particular, internal segments belonging to subtypes H1–H4 reassort with a lower rate compared with H5 and H9. It should be noted that this dataset included poultry AIV and poultry-outbreak-related AIV sequences, likely influencing reassortment rates (Lu *et al.*, 2014).

Overall genetic diversity of AIVs in Eurasian Anseriformes can be captured by the genetic diversity found in dabbling ducks. AIVs isolated from dabbling ducks in Alberta are a good representation of the genetic diversity of AIVs circulating in North America (Bahl *et al.*, 2013). In contrast, AIVs from West Eurasia, East Eurasia, Central Eurasia or Oceania do not represent the genetic diversity of the whole of Eurasia well. The genetic diversity of AIVs is shaped by many factors such as immunogenicity of the host, reassortment, migration patterns and life span of the hosts as well as virus durability in aquatic environments (Roche *et al.*, 2014). The influence of heterosubtypic immunity is seen on the prevalence of both HA groups and on the level of HA clades in recaptured wild ducks (Latorre-Margalef *et al.*, 2013). The incidence and prevalence of AIVs shows clear seasonal patterns due to host–pathogen interactions. The influx of immunologically naïve juveniles in summer and the arrival of susceptible migrants in autumn as well as moult aggregations are also likely drivers of AIV infection dynamics in temperate Eurasian latitudes (Jehl, 1990; van Dijk *et al.*, 2014). Whether these disease dynamics patterns can be generalized over multiple subpopulations in different latitudes within Eurasia remains to be investigated. In some North American flyways, resident birds can also act as reservoirs of virus diversity and although migratory birds introduce AIV in these wild bird populations, these viruses do not necessarily become the predominantly circulating viruses (Hill *et al.*, 2012). Whilst this might be true at sites in Eurasia where resident and migratory bird populations overlap, in many areas there is likely less opportunity for resident maintenance. Therefore, virus diversity is more likely driven by migration.

Here, we map the long-term spatial-temporal dynamics of the whole-genome of AIV in Eurasia. Despite in-depth wild bird surveillance in Eurasia, it is clear from this study that to assess the implication of migration patterns on the genetic diversity of AIV in Eurasia future whole-genome sequencing should be directed towards increased numbers of samples within a short time frame in locations along the different flyways. Such high-resolution studies have been performed in North America and West Eurasia, and are currently being actively pursued in the rest of Eurasia. Incorporating of metadata such as host species, location and date of sampling, age, sex, and migratory status will illuminate future host-focused studies by including the impact of ecological factors like individual species diversity and life cycle on AIV genetic diversity.

## Methods

### Dataset and genomic sequencing

Over a period of 15 years, 186 054 samples from 440 different bird species were analysed for the presence of AIVs. Positive isolates were subtyped and sequenced. In collaboration with the National Institutes of Health and the J. Craig Venter Institute, ∼83 full or nearly full genomes and 30 partial genomes of AIVs have been submitted to GenBank.

The coding complete genomes of the influenza viruses were sequenced using a high-throughput next-generation sequencing pipeline at the J. Craig Venter Institute, which included the 454/Roche GS-FLX and the Illumina HiSeq 2000 platforms. Viral RNA was isolated using a ZR 96 Viral RNA kit (Zymo Research). The influenza A genomic RNA segments were simultaneously amplified from 3 μl purified RNA using a multisegment reverse transcription (M-RT)-PCR strategy (Zhou *et al.*, 2009; Zhou & Wentworth, 2012) The influenza M-RT-PCR amplicons were barcoded and amplified using an optimized SISPA (sequence-independent single primer amplification) protocol (Djikeng *et al.*, 2008, 2009). Subsequently, the SISPA amplicons were purified, pooled and size selected (∼800 or∼200 bp), and the pools were used for both Roche 454 (Roche Diagnostics) and Illumina (Illumina) library construction. Samples were sequenced on the 454/Roche GS-FLX and Illumina HiSeq 2000 platforms. Libraries were prepared for sequencing on the 454/Roche GS-FLX platform using Titanium chemistry or for sequencing on the Illumina HiSeq 2000. The sequence reads were sorted by barcode, trimmed and searched by tblastx against custom nucleotide databases of full-length influenza A segments downloaded from GenBank to filter out both chimeric influenza sequences and non-influenza sequences amplified during the random hexamer-primed amplification. The reads were binned by segment and the 454/Roche GS-FLX reads were *de novo* assembled using the clc_novo_assemble program (CLC Bio). The resulting contigs were searched against the corresponding custom full-length Influenza segment nucleotide database to find the closest reference sequence for each segment. Both 454/Roche GS-FLX and Illumina HiSeq 2000 reads were then mapped to the selected reference influenza A virus segments using the clc_ref_assemble_long program (CLC Bio). At loci where both 454/Roche GS-FLX and Illumina HiSeq 2000 sequence data agreed on a variation (as compared with the reference sequence), the reference sequence was updated to reflect the difference. A final mapping of all next-generation sequences to the updated reference sequences was then performed. Any regions of the viral genomes that were poorly covered or ambiguous after next-generation sequencing were amplified and sequenced using the standard Sanger sequencing approach.

These viruses were isolated from different wild bird species, and included different subtypes and sampling locations within West Eurasia throughout the time period of the study. In addition, all full-genome sequences from AIV genomes containing NA1–NA9 and HA1–HA12 available from GenBank were retrieved. All sequences from domestic birds and all sequences related to poultry outbreaks, particularly HPAI H5N1, H7 and H9, were excluded. Our final datasets of matched genome sequences for PB2 (2266 nt), PB1 (2259 nt), PA (2142 nt), HA (1716 nt), NP (1482 nt), NA (1374 nt), MP (979 nt) and NS (838 nt) were aligned with BioEdit version 7.1 (a total of 211 complete genomes; see Table S1 for GenBank accession numbers).

### ML analysis

Phylogenetic trees for each segment were reconstructed with PhyML version 3.0 (Guindon & Gascuel, 2003), using the general time reversible (GTR) nucleotide substitution model with a proportion of invariant sites and a Γ distribution of among-site rate variation, all estimated from the data (determined by ModelTest as the appropriate nucleotide substitution model). garli version 0.96 (Zwickl, 2006) was run on the best tree from PhyML for 1 million generations to optimize tree topology and branch lengths.

### Temporal phylogeny and relative genetic diversity

To identify potential errors in sequence data annotation that might have affected the clock estimation, we used the reconstructed ML nucleotide trees in Path-O-Gen version 1.3 (http://tree.bio.ed.ac.uk/software/pathogen) to generate linear regression plots of the years of sampling versus root-to-tip distance. We did not observe any anomalies in the eight segment datasets, which all exhibited a clock-like behaviour (Drummond *et al.*, 2003).

We estimated rates of evolutionary change (nucleotide substitutions per site per year) and times of circulation of the MRCA (years) with beast version 1.7.3 using time-stamped sequence data with a relaxed-clock Bayesian Markov chain Monte Carlo (MCMC) method (Drummond & Rambaut, 2007; Drummond *et al.*, 2005, 2006). For all analyses, the uncorrelated log-normal relaxed molecular clock and a Γ site heterogeneity model with four Γ categories was used in combination with the GTR nucleotide substitution model. A normal rate prior with a mean of 0.0033 substitutions per site per year (sd = 0.0016) was used (Bahl *et al.*, 2011). These analyses were conducted with a Bayesian Skyline coalescent model, a random starting tree and a constant rate of migration. We performed at least three independent analyses of at least 100 million MCMC chains to ensure convergence and combined these analyses after removal of the burn-in of 10 % using LogCombiner version 1.7.3. Finally, the MCMC chains were summarized to reconstruct the MCC trees using TreeAnnotator version 1.7.3. Trees were visualized and coloured with the FigTree program version 1.4.0 (http://tree.bio.ed.ac.uk/software/figtree/).

### Phylogeography

We grouped our country-level dataset into West Eurasia, Central Eurasia, East Eurasia and Oceania because of insufficient sampling density to reconstruct exact sampling location of ancestral viruses. Discrete state ancestral reconstruction of viral sampling locations and migration rates between geographical regions were estimated with an asymmetrical state transition model. Given the large number of states, a Bayesian stochastic search variable selection (BSSVS) was employed to reduce the number of parameters to those with significantly non-zero transition rates (Lemey *et al.*, 2009). From the BSSVS results, a Bayes factor (BF) test could be applied to assess the support for individual transitions between discrete geographical states. The BF was deemed statistically significant if the combined independent analyses resulted in a binary indicator >0.5 and a BF>6. Therefore, our minimal critical cut-offs for statistical support were 8 ≤ BF ≤ 100 indicating support and BF>100 indicating strong support (Bahl *et al.*, 2011; Lemey *et al.*, 2009).

The migration routes that had the strongest support as indicated by the highest BF (Lemey *et al.*, 2009) were determined using spread (Bielejec *et al.*, 2011). In addition, significant clustering of sequences from the four geographical regions was investigated by coding the regional location from which the virus was sampled onto the tips of 900 posterior sampled trees, generating 100 null distributions, and testing the significance of the observed data using BaTS package (Parker *et al.*, 2008). *P* < 0.05 indicated significant clustering, whilst *P*>0.05 indicated that traits were randomly distributed across the phylogeny.

### Reassortment analyses

To visualize similarities and differences between the phylogenies, and investigate reassortment, tanglegrams were generated using the nucleotide substitution MCC trees generated by beast and TreeMap version 1.0 (http://taxonomy.zoology.gla.ac.uk/rod/treemap.html). These tanglegrams consisted of two rooted phylogenetic trees of which taxa that corresponded to each other in the two trees were connected. In the absence of reassortment, one would expect to see nearly horizontal linkage connecting one taxa to another.

## Supplementary Data

1996 Supplementary Data
