# Supplementary material for: Influenza A virus evolution and spatio-temporal dynamics in Eurasian wild birds: a phylogenetic and phylogeographical study of whole-genome sequence data
Source: J Gen Virol. 2015 Aug;96(Pt 8):2050–60. doi: 10.1099/vir.0.000155 (PMC4681060; doi:10.1099/vir.0.000155)

Table S1. Accession numbers

	1(PB2)*	2(PB1)	3(PA)	4(HA)	5(NP)	6(NA)	7(M)	8(NS)
A/Anascrecca/Spain/1460/08	HQ244404	HQ244405	HQ244406	HQ244407	HQ244408	HQ244409	HQ244410	HQ244411
A/Anasplatyrhynchos/Belgium/12827/07	HM570065	HM570064	HM570063	HM570058	HM570061	HM570060	HM570059	HM570062
A/barheadedgoose/Mongolia/143/05	GQ907293	GQ907292	GQ907291	GQ907286	GQ907289	GQ907288	GQ907287	GQ907290
A/barnaclegoose/NL/1/05	**CY041393**	**CY041392**	**CY041391**	**CY041386**	**CY041389**	**CY041388**	**CY041387**	**CY041390**
A/Bewickswan/NL/1/07	**CY076983**	**CY076982**	**CY076981**	**CY076976**	**CY076979**	**CY076978**	**CY076977**	**CY076980**
A/Bewickswan/NL/5/07	**CY041281**	**CY041280**	**CY041279**	**CY041274**	**CY041277**	**CY041276**	**CY041275**	**CY041278**
A/blackduck/AUS/4045/80	CY005698	CY005697	CY005696	CY005691	CY005694	CY005693	CY005692	CY005695
A/blackduck/AUS/751/78	CY005659	CY005658	CY005657	CY006031	CY005655	CY005654	CY005653	CY005656
A/blackduck/Perth/699/78	CY028643	CY028642	CY028641	CY028636	CY028639	CY028638	CY028637	CY028640
A/blackduck/WesternAus/4954/83	CY028274	CY028273	CY028272	CY028267	CY028270	CY028269	CY028268	CY028271
A/blackduck/WesternAus/702/78	CY041313	CY041312	CY041311	CY041306	CY041309	CY041308	CY041307	CY041310
A/black-headedgull/NL/1/05	**CY041385**	**CY041384**	**CY041383**	**CY041378**	**CY041381**	**CY041380**	**CY041379**	**CY041382**
A/black-headedgull/NL/1/06	**CY076999**	**CY076998**	**CY076997**	**CY076992**	**CY076995**	**CY076994**	**CY076993**	**CY076996**
A/commoneider/NL/1/06	**CY077007**	**CY077006**	**CY077005**	**CY077000**	**CY077003**	**CY077002**	**CY077001**	**CY077004**
A/commoneider/NL2/06	**CY041345**	**CY041344#**	**CY041343**	**CY041338**	**CY041341**	**CY041340**	**CY041339**	**CY041342**
A/commonteal/NL/1/05	**CY041265**	**CY041264**	**CY041263**	**CY041258**	**CY041261**	**CY041260**	**CY041259**	**CY041262**
A/commonteal/NL/10/00	**CY060175**	**CY060176**	**CY060177**	**CY060178**	**CY060179**	**CY060180**	**CY060181**	**CY060182**
A/commonteal/NL/2/05	**CY041377**	**CY041376**	**CY041375**	**CY041370**	**CY041373**	**CY041372**	**CY041371**	**CY041374**
A/commonteal/NL/9/00	**CY060187**	**CY060188**	**CY060189**	**CY060190**	**CY060191**	**CY060192**	**CY060193**	**CY060194**
A/duck/Altai/1285/1991	GQ227557	GQ227558	GQ227554	GQ227551	GQ227553	GQ227552	GQ227555	GQ227556
A/duck/Aus/749/80	CY005690	CY005689	CY005688	CY014627	CY005687	CY005686	CY014628	CY014629
A/duck/Beijing/40/04	EU492488	EU492494	EU492500	EU492530	EU492512	EU492518	EU492524	EU492506
A/duck/Beijing/61/05	EU492492	EU492498	EU492504	EU492534	EU492516	EU492522	EU492528	EU492510
A/duck/Chabarovsk/1610/1972	CY014709	CY014708	CY014707	CY014702	CY014705	CY014704	CY014703	CY014706
A/duck/CzechRepublic/1/1956	CY045334	CY045333	CY045332	CY045327	CY045330	CY045329	CY045328	CY045331
A/duck/Denmark/53-147-8/08	GQ401162	GQ401163	GQ401164	GQ401157	GQ401159	GQ401158	GQ401160	GQ401161
A/duck/Denmark/65047/04	DQ251449	DQ251450	DQ251451	DQ251447	DQ251452	DQ251448	DQ251453	DQ251454
A/duck/England/1/1956	CY014686	CY014685	CY014684	CY014679	CY014682	CY014681	CY014680	CY014683
A/duck/France/02166/02	CY046117	CY046118	CY046119	AJ632268	CY046120	AJ849934	AM040281	CY046121
A/duck/France/05066b/05	CY046134	CY046135	CY046136	AJ971297	CY046137	AJ972921	AJ973609	CY046138
A/duck/France/06436/06	CY046147	CY046148	CY046149	CY046150	CY046151	CY046152	CY046153	CY046154
A/duck/France/080032/08	CY046171	CY046172	CY046173	CY046174	CY046175	CY046176	CY046177	CY046178
A/duck/France/080036/08	CY046179	CY046180	CY046181	CY046182	CY046183	CY046184	CY046185	CY046186
A/duck/Hokkaido/49/1998	AB473937	AB473938	AB473939	AB125928	AB473940	AB251944	AB473941	AB473942
A/duck/Hokkaido/8/80	AB274963	AB274964	AB274965	AB275283	AB275284	AB275285	AB275286	AB275287
A/duck/Hokkaido/9/99	AB262460	AB262461	AB262462	AB262463	AB262464	AB262465	AB262466	AB262467
A/duck/HongKong/147/77	CY005646	CY005645	CY005645	CY005639	CY005642	CY005641	CY005640	CY005643
A/duck/HongKong/205/77	CY005596	CY005595	CY005594	CY014615	CY005592	CY005591	CY005590	CY005593
A/duck/HongKong/24/1976	CY005631	CY005630	CY005629	CY006030	CY005627	CY005626	CY005625	CY005628
A/duck/HongKong/278/78	CY014614	CY005552	CY005551	CY005546	CY005549	CY005548	CY005547	CY005550
A/duck/HongKong/319/78	CY005545	CY005544	CY005543	CY005538	CY005541	CY005540	CY005539	CY005542
A/duck/HongKong/342/78	CY005582	CY005581	CY005580	CY005575	CY005578	CY005577	CY005576	CY005579
A/duck/HongKong/365/78	CY005574	CY005573	CY005572	CY006027	CY005570	CY005569	CY005568	CY005571
A/duck/HongKong/562/79	CY005652	CY005651	CY005650	CY014619	CY005648	CY014620	CY005647	CY005649
A/duck/HongKong/7/75	CY005559	CY005558	CY005557	CY006026	CY005555	CY005554	CY005553	CY005556
A/duck/HongKong/702/79	CY031256	CY031257	CY031258	CY031259	CY031260	CY031261	CY031262	CY031263
A/duck/HongKong/784/79	CY005638	CY005637	CY005636	CY005632	CY005634	CY014618	CY005633	CY005635
A/duck/Hunan/8-19/09	HQ285883	HQ285884	HQ285885	HQ285886	HQ285887	HQ285888	HQ285889	HQ285890
A/duck/Italy/194659/06	FJ432769	FJ432768	FJ432767	FJ432762	FJ432765	FJ432764	FJ432763	FJ432766
A/duck/Italy/281904/06	FJ432777	FJ432776	FJ432775	FJ432770	FJ432773	FJ432772	FJ432771	FJ432774
A/duck/Italy/69238/07	FJ432761	FJ432760	FJ432759	FJ432754	FJ432757	FJ432756	FJ432755	FJ432758
A/duck/Italy/775/04	CY024753	CY024752	CY024751	CY024746	CY024749	CY024748	CY024747	CY024750
A/duck/Malaysia/F118-08-04/04	EU249545	EU249546	EU249547	DQ104701	EU249548	DQ104703	EU249550	EU249549
A/duck/Mongolia/47/01	AB473548	AB268552	AB268553	AB268557	AB268554	AB302788	AB268555	AB268556
A/duck/Mongolia/867/02	AB473540	AB473541	AB473542	AB473543	AB473544	AB473545	AB473546	AB473547
A/duck/Nanchang/1681/1992	CY005475	CY005474	CY005473	CY006016	CY005471	CY005470	CY005469	CY005472
A/duck/Nanchang/1904/1992	CY005500	CY005499	CY005498	CY014612	CY005496	CY005495	CY005494	CY005497
A/duck/Nanchang/2-0486/00	CY005437	CY005436	CY005435	CY014608	CY005433	CY005432	CY005431	CY005434
A/duck/Nanchang/4-165/00	CY005493	CY005492	CY005491	CY006017	CY005489	CY005488	CY005487	CY005490
A/duck/Nanchang/4-184/00	CY005444	CY005443	CY005442	CY014609	CY005440	CY005439	CY005438	CY005441
A/duck/Potsdam/1402-6/1986	CY005783	CY005782	CY005781	CY014642	CY005779	CY005778	CY005777	CY005780
A/duck/Potsdam/2216-4/84	CY005776	CY005775	CY005774	CY006036	CY005772	CY005771	CY005770	CY005773
A/duck/Primorie/2621/01	GQ162793	GQ162792	GQ162791	GQ162786	GQ162790	GQ162788	GQ162787	GQ162789
A/duck/Primorie/2633/01	GQ227610	GQ227611	GQ227607	GQ227604	GQ227606	GQ227605	GQ227608	GQ227609
A/duck/Shiga/8/04	AB304144	AB304145	AB304146	AB304147	AB304148	AB304149	AB304150	AB304151
A/duck/Taiwan/A68/03	DQ376898	DQ376862	DQ376826	DQ376646	DQ376754	DQ376718	DQ376682	DQ376790
A/duck/Taiwan/WB29/99	DQ376879	DQ376843	DQ376807	DQ376627	DQ376735	DQ376699	DQ376663	DQ376771
A/duck/Taiwan/WB459/04	DQ376903	DQ376867	DQ376831	DQ376651	DQ376759	DQ376723	DQ376687	DQ376795
A/duck/Tasmania/277/07	CY033168	CY033167	CY033166	CY033161	CY033164	CY033163	CY033162	CY033165
A/duck/Ukraine/1/1963	CY005819	CY005818	CY005817	CY006038	CY005815	CY014648	CY005814	CY005816
A/duck/Victoria/512/07	CY061617	CY061616	CY061615	CY061610	CY061613	CY061612	CY061611	CY061614
A/duck/Victoria/5384/02	CY028258	CY028257	CY028256	CY028251	CY028254	CY028253	CY028252	CY028255
A/duck/Victoria/D23/1994	CY045254	CY045253	CY045252	CY045247	CY045250	CY045249	CY045248	CY045251
A/duck/Yangzhou/02/05	EF061121	EF061124	EF061120	EF061122	EF061123	EF061126	EF061125	EF061119
A/dunlin/Sweden/1/05	**CY076991**	**CY076990**	**CY076989**	**CY076984**	**CY076987**	**CY076986**	**CY076985**	**CY076988**
A/eurasiancoot/WesternAus/2727/79	CY028250	CY028249	CY028248	CY028243	CY028246	CY028245	CY028244	CY028247
A/Eurasianwigeon/NL/3/05	**CY043863**	**CY043862**	**CY043861**	**CY043856**	**CY043859**	**CY043858**	**CY043857**	**CY043860**
A/Eurasianwigeon/NL/4/07	**CY077055**	**CY077054**	**CY077053**	**CY077048**	**CY077051**	**CY077050**	**CY077049**	**CY077052**
A/Eurasianwigeon/Sweden/1/04	**CY041369**	**CY041368**	**CY041367**	**CY041362**	**CY041365**	**CY041364**	**CY041363**	**CY041366**
A/fowl/Hampshire/PD378/1985	GQ176113	GQ176114	GQ176115	GQ176120	GQ176117	GQ176118	GQ176119	GQ176116
A/gadwall/Altai/1202/07	CY049753	CY049754	CY049755	CY049756	CY049757	CY049758	CY049759	CY049760
A/gadwall/Altai/1324/07	CY049785	CY049786	CY049787	CY049788	CY049789	CY049790	CY049791	CY049792
A/gadwall/Altai/1326/07	CY049801	CY049802	CY049803	CY049804	CY049805	CY049806	CY049807	CY049808
A/gadwall/Altai/1328/07	CY049809	CY049810	CY049811	CY049812	CY049813	CY049814	CY049815	CY049816
A/gadwall/NL/1/06	**CY043871**	**CY043870**	**CY043869**	**CY043864**	**CY043867**	**CY043866**	**CY043865**	**CY043868**
A/garganey/Altai/1213/07	CY049769	CY049770	CY049771	CY049772	CY049773	CY049774	CY049775	CY049776
A/goose/HongKong/23/78	CY005589	CY005588	CY005587	CY006028	CY005585	CY005584	CY005583	CY005586
A/goose/Italy/296426/03	FJ432785	FJ432784	FJ432783	FJ432778	FJ432781	FJ432780	FJ432779	FJ432782
A/grayteal/Aus/1/79	CY005671	CY005670	CY005669	CY014623	CY005667	CY014624	CY005666	CY005668
A/grayteal/Aus/3/79	CY005685	CY005684	CY005684	CY005679	CY005682	CY005681	CY005680	CY005683

A/grayteal/WesternAus/1840/79	CY045270	CY045269	CY045268	CY045263	CY045266	CY045265	CY045264	CY045267
A/grayteal/WesternAus/1855/79	CY031163	CY031162	CY031161	CY031156	CY031159	CY031158	CY031157	CY031160
A/greylaggoose/NL/4/99	CY060195	CY060196	CY060197	CY060198	CY060199	CY060200	CY060201	CY060202
A/gull/Moscow/3100/06	EU152234	EU152235	EU152236	EU152237	EU152238	EU152239	EU152240	EU152241
A/herringgull/NL/4/06	CY077039	CY077038	CY077037	CY077032	CY077035	CY077034	CY077033	CY077036
A/mallard/Altai/1208/07	CY049761	CY049762	CY049763	CY049764	CY049765	CY049766	CY049767	CY049768
A/mallard/Bavaria/185-26/08	HQ259229	HQ259230	HQ259231	HQ259232	HQ259233	HQ259234	HQ259235	HQ259236
A/mallard/Bavaria/185-8/08	HQ259221	HQ259222	HQ259223	HQ259224	HQ259225	HQ259226	HQ259227	HQ259228
A/mallard/CzechRepublic/15902-17K/09	HQ244427	HQ244428	HQ244429	HQ244430	HQ244431	HQ244432	HQ244433	HQ244434
A/mallard/Denmark/64650/03	DQ251441	DQ251442	DQ251443	HE802063	DQ251444	DQ251445	AY531030	DQ251446
A/mallard/France/061054/06	CY046139	CY046140	CY046141	CY046142	CY046143	CY046144	CY046145	CY046146
A/mallard/France/06964/06	CY046155	CY046156	CY046157	CY046158	CY046159	CY046160	CY046161	CY046162
A/mallard/Gloucestershire/PD374/1985	GQ176121	GQ176122	GQ176123	GQ176128	GQ176125	GQ176126	GQ176127	GQ176124
A/mallard/Hokkaido/24/09	AB530989	AB530990	AB530991	AB530992	AB530993	AB530994	AB530995	AB530996
A/mallard/Italy/3401/05	CY021404	CY021403	CY021402	CY021397	CY021400	CY021399	CY021398	CY021401
A/mallard/Italy/4223-2/06	CY034765	CY034764	CY034763	CY034758	CY034761	CY034760	CY034759	CY034762
A/mallard/Marquenterre/Z237/83	DQ864507	DQ864506	DQ864508	GU066779	DQ864509	GU066780	GU066781	GU066782
A/mallard/NL/02/00	CY076952	CY076951	CY076950	CY076945	CY076948	CY076947	CY076946	CY076949
A/mallard/NL/1/99	CY060238	CY060239	CY060240	CY060241	CY060242	CY060243	CY060244	CY060245
A/mallard/NL/1/06	CY043855	CY043854	CY043853	CY043848	CY043851	CY043850	CY043849	CY043852
A/mallard/NL/1/07	CY043823	CY043822	CY043821	CY043816	CY043819	CY043818	CY043817	CY043820
A/mallard/NL/11/07	CY041409	CY041408	CY041407	CY041402	CY041405	CY041404	CY041403	CY041406
A/mallard/NL/12/00	KF695236	KF695237	KF695238	KF695239	KF695240	KF695241	KF695242	KF695243
A/mallard/NL/12/01	CY060217	CY060218	CY060219	CY060220	CY060221	KF695235	CY060222	CY060223
A/mallard/NL/14/07	CY041241	CY041240	CY041239	CY041234	CY041237	CY041236	CY041235	CY041238
A/mallard/NL/15/99	CY060224	CY060225	CY060226	CY060227	CY060228	CY189928	CY060229	CY060230
A/mallard/NL/17/07	CY043887	CY043886	CY043885	CY043880	CY043883	CY043882	CY043881	CY043884
A/mallard/NL/2/99	CY060258	CY060259	CY060260	CY060261	CY060262	CY060263	CY060264	CY060265
A/mallard/NL/2/05	CY041257	CY041256	CY041255	CY041250	CY041253	CY041252	CY041251	CY041254
A/mallard/NL/22/07	CY043847	CY043846	CY043845	CY043840	CY043843	CY043842	CY043841	CY043844
A/mallard/NL/26/05	CY041425	CY041424	CY041423	CY041418	CY041421	CY041418	CY041419	CY041422
A/mallard/NL/28/06	CY076912	CY076911	CY076910	CY076905	CY076908	CY076907	CY076906	CY076909
A/mallard/NL/29/06	CY043839	CY043838	CY043837	CY043832	CY043835	CY043834	CY043833	CY043836
A/mallard/NL/3/99	CY064939	CY064940	CY064941	CY064942	CY064943	CY064944	CY064945	CY064946
A/mallard/NL/3/05	CY041249	CY041248	CY041247	CY041242	CY041245	CY041244	CY041243	CY041246
A/mallard/NL/30/06	CY076904	CY076903	CY076902	CY076897	CY076900	CY076899	CY076898	CY076901
A/mallard/NL/31/06	CY041233	CY041232	CY041231	CY041226	CY041229	CY041228	CY041227	CY041230
A/mallard/NL/33/06	CY041417	CY041416	CY041415	CY041410	CY041413	CY041412	CY041411	CY041414
A/mallard/NL/4/06	CY076959	CY076958	CY076957	CY077064	CY076955	CY076954	CY076953	CY076956
A/mallard/NL/42/06	CY077063	CY077062	CY077061	CY077056	CY077059	CY077058	CY077057	CY077060
A/mallard/NL/5/99	CY064947	CY064948	CY064949	CY064950	CY064951	CY064952	CY064953	CY064954
A/mallard/NL/5/01	CY060340	CY060341	CY060342	CY060343	CY060344	KF695299	CY060345	CY060346
A/mallard/NL/65/06	CY076944	CY076943	CY076942	CY076937	CY076940	CY076939	CY076938	CY076941
A/mallard/NL/71/06	CY041401	CY041400	CY041399	CY041394	CY041397	CY041396	CY041395	CY041398
A/mallard/NL/9/05	CY077015	CY077014	CY077013	CY077008	CY077011	CY077010	CY077009	CY077012
A/mallard/NewZealand/1365-350/05	CY077591	CY077590	CY077589	CY077584	CY077587	CY077586	CY077585	CY077588
A/mallard/NewZealand/1615-17/04	CY045366	CY045365	CY045364	CY045359	CY045362	CY045361	CY045360	CY045363
A/mallard/Postdam/178-4/83	DQ017501	DQ017500	DQ017499	DQ017496	DQ017497	DQ017496	DQ017494	DQ017498
A/mallard/PT/35910-2/06	HM849024	HM849023	HM849022	HM849017	HM849020	HM849019	HM849018	HM849021
A/Mallard/Sweden/102/02	KF695282	KF695283	KF695284	KF695285	KF695286	KF695287	KF695288	KF695289
A/mallard/Sweden/105/02	KF695333	KF695334	KF695335	KF695336	KF695337	KF695338	KF695339	KF695340
A/mallard/Sweden/21/02	KF695267	KF695268	KF695269	KF695270	KF695271	KF695272	KF695273	KF695274
A/mallard/Sweden/24/02	CY060246	CY060247	CY060248	CY060249	KF695204	CY064796	CY060250	CY060251
A/mallard/Sweden/30/05	CY043831	CY043830	CY043829	CY043824	CY043827	CY043826	CY043825	CY043828
A/mallard/Sweden/4/05	CY043879	CY043878	CY043877	CY043872	CY043875	CY043874	CY043873	CY043876
A/mallard/Sweden/45/02	CY060278	CY060279	CY060280	CY060281	CY060282	CY060283	CY060284	KF695311
A/mallard/Sweden/45/05	CY041337	CY041336	CY041335	CY041330	CY041333	CY041332	CY041331	CY041334
A/mallard/Sweden/46/02	CY060285	CY060286	CY060287	CY060288	CY060289	KF695218	CY060290	KF695219
A/mallard/Sweden/48/02	CY060291	CY060292	CY060293	CY060294	CY060295	CY060296	CY060297	CY060298
A/mallard/Sweden/49/02	KF695325	KF695326	KF695327	KF695328	KF695329	KF695330	KF695331	KF695332
A/mallard/Sweden/50/02	CY060305	CY060306	CY060307	CY060308	CY060309	CY060310	CY060311	CY060312
A/mallard/Sweden/52/03	CY060313	CY060314	CY060315	CY060316	CY060317	CY060318	CY060319	CY060320
A/Mallard/Sweden/54/02	KF695341	KF695342	KF695343	KF695344	KF695345	CY189927	KF695346	KF695347
A/Mallard/Sweden/54/03	KF695220	KF695221	KF695222	KF695223	KF695224	KF695225	KF695226	KF695227
A/mallard/Sweden/55/02	CY060321	CY060322	CY064797	CY060323	CY060324	CY060325	CY060326	CY060327
A/mallard/Sweden/56/02	KF695351	KF695352	KF695353	KF695354	KF695355	KF695356	KF695357	KF695358
A/mallard/Sweden/58/03	CY060332	CY060333	CY060334	CY060335	CY060336	CY060337	CY060338	CY060339
A/mallard/Sweden/6/02	CY060363	CY060364	CY060365	CY060366	CY060367	CY060368	CY060369	CY060370
A/mallard/Sweden/62/03	CY060347	CY060348	CY060349	CY060350	CY060351	CY060352	CY060353	CY060354
A/mallard/Sweden/65/02	CY060355	CY060356	CY060357	CY060358	CY060359	CY060360	CY060361	CY060362
A/mallard/Sweden/65/05	CY041361	CY041360	CY041359	CY041354	CY041357	CY041356	CY041355	CY041358
A/mallard/Sweden/7/02	KF695300	KF695301	KF695302	KF695303	KF695304	KF695305	KF695306	KF695307
A/mallard/Sweden/7/03	CY060371	CY060372	CY060373	CY060374	CY060375	CY060376	CY060377	CY060378
A/mallard/Sweden/74/03	CY076936	CY076935	CY076934	CY076929	CY076932	CY076931	CY076930	CY076933
A/mallard/Sweden/81/02	CY060379	CY060380	CY060381	CY060382	CY060383	CY060384	CY060385	CY060386
A/mallard/Sweden/85/02	KF695290	KF695291	KF695292	KF695293	KF695294	KF695295	KF695296	KF695297
A/Mallard/Sweden/91/02	KF695251	KF695252	KF695253	KF695254	KF695255	KF695256	KF695257	KF695258
A/mallard/Sweden/95/05	CY077023	CY077022	CY077021	CY077016	CY077019	CY077018	CY077017	CY077020
A/mallard/Yanchen/05	EU880349	EU880348	EU880347	EU880342	EU880345	EU880344	EU880343	EU880346
A/mallard/ZhaLong/88/04	FJ349254	FJ349253	FJ349252	FJ349247	FJ349249	FJ349248	FJ349251	FJ349250
A/muteswan/NL/1/06	CY077047	CY077046	CY077045	CY077040	CY077043	CY077042	CY077041	CY077044
A/northernpintail/Akita/714/06	AB490823	AB490824	AB490825	AB490826	AB490827	AB490828	AB490829	AB490830
A/northernpintail/Sweden/1/03	CY060421	CY060422	CY060423	CY060424	CY060425	CY060426	CY060427	KF695298
A/northernshoveler/NL/1/06	CY077031	CY077030	CY077029	CY077024	CY077027	CY077026	CY077025	CY077028
A/northernshoveler/NL/18/99	CY060414	CY060415	CY060416	CY060417	CY060418	CY189928	CY060419	CY060420
A/pink-footedgoose/NL/1/06	CY041273	CY041272	CY041271	CY041266	CY041269	CY041268	CY041267	CY041270
A/redcrestedpochard/Mongolia/1915/06	GQ907333	GQ907332	GQ907331	GQ907326	GQ907329	GQ907328	GQ907327	GQ907330
A/red-neckedstint/Aus/2/04	CY028266	CY028265	CY028264	CY028259	CY028262	CY028261	CY028260	CY028263
A/red-neckedstint/Aus/3/04	CY034749	CY034748	CY034747	CY034742	CY034745	CY034744	CY034743	CY034746
A/red-neckedstint/Aus/4189/80	CY014632	CY005703	CY005702	CY014630	CY005700	CY014631	CY005699	CY005701
A/red-neckedstint/Aus/4500/80	CY014635	CY005708	CY005707	CY014633	CY005705	CY014634	CY005704	CY005706
A/red-neckedstint/Aus/5/04	CY029896	CY029895	CY029894	CY029889	CY029892	CY029891	CY029890	CY029893
A/red-neckedstint/Aus/5745/81	CY005715	CY005714	CY005713	CY014636	CY005711	CY005710	CY005709	CY005712
A/red-neckedstint/Bunbury/4500/80	CY028282	CY028281	CY028280	CY028275	CY028278	CY028277	CY028276	CY028279

A/red-neckedstint/WesternAus/4094/84	CY035897	CY035896	CY035895	CY035890	CY035893	CY035892	CY035891	CY035894
A/red-neckedstint/WesternAus/4126/80	CY033160	CY033159	CY033158	CY033153	CY033156	CY033155	CY033154	CY033157
A/red-neckedstint/WesternAus/4915/84	CY029904	CY029903	CY029902	CY029897	CY029900	CY029899	CY029898	CY029901
A/red-neckedstint/WesternAus/4923/83	CY028290	CY028289	CY028288	CY028283	CY028286	CY028285	CY028284	CY028287
A/red-neckedstint/WesternAus/5745/82	CY029880	CY029879	CY029878	CY029873	CY029876	CY029875	CY029874	CY029877
A/ruddyshelduck/Mongolia/37/05	GQ907341	GQ907340	GQ907339	GQ907334	GQ907337	GQ907336	GQ907335	GQ907338
A/ruddyshelduck/Mongolia/P52/05	GQ907349	GQ907348	GQ907347	GQ907342	GQ907345	GQ907344	GQ907343	GQ907346
A/sharp-tailedsandpiper/Aus/10/04	CY029888	CY029887	CY029886	CY029881	CY029884	CY029883	CY029882	CY029885
A/sharp-tailedsandpiper/Aus/6/04	CY025204	CY025203	CY025202	CY025197	CY025200	CY025199	CY025198	CY025201
A/shearwater/Aus/1/1973	CY005827	CY005826	CY005825	CY014656	CY014658	CY014657	CY005823	CY005824
A/shearwater/Aus/405/78	CY005665	CY005664	CY005663	CY014621	CY005661	CY014622	CY005660	CY005662
A/shearwater/Aus/751/75	CY045262	CY045261	CY045260	CY045255	CY045258	CY045257	CY045256	CY045259
A/shoveler/NL/19/99	CY005858	CY005857	CY005856	CY014719	CY005854	CY005853	CY005852	CY005855
A/slaty-backedgull/Japan/6KS0185/06	CY079298	CY079297	CY079296	CY079291	CY079294	CY079293	CY079292	CY079295
A/spotbillduck/Xuyi/6/05	GQ203122	GQ203123	GQ203124	GQ184327	GQ169500	GQ184332	GQ219713	GQ219714
A/swan/Slovenia/53/09	HQ283354	HQ283355	HQ283356	HQ283357	HQ283358	HQ283359	HQ283360	HQ283361
A/teal/Germany/WV632/05	CY061882	CY061883	CY061884	CY061885	CY061886	CY061887	CY061888	CY061889
A/teal/Italy/3812/05	CY022652	CY022651	CY022650	CY022645	CY022648	CY022647	CY022646	CY022649
A/teal/Italy/3931-38/05	CY022644	CY022643	CY022642	CY022637	CY022640	CY022639	CY022638	CY022641
A/tern/Aus/752/75	CY077659	CY077658	CY077657	CY077650	CY077655	CY077654	CY077653	CY077656
A/tuftedduck/PT/13771/06	HM849010	HM849009	HM849008	HM849003	HM849006	HM849005	HM849004	HM849007
A/turnstone/NL/1/07	**CY041353**	**CY041352**	**CY041351**	**CY041346**	**CY041349**	**CY041348**	**CY041347**	**CY041350**
A/wedge-tailedshearwater/WesternAus/405/77	CY028659	CY028658	CY028657	CY028652	CY028655	CY028654	CY028653	CY028656
A/whistlingswan/Shimane/468/88	GQ176105	GQ176106	GQ176107	GQ176112	GQ176109	GQ176110	GQ176111	GQ176108
A/whitefrontedgoose/NL/1/99	**CY060428**	**CY060429**	**CY060430**	**CY060431**	**CY060432**	**CY060433**	**CY060434**	**CY060435**
A/whitefrontedgoose/NL/2/99	**CY060436**	**CY060437**	**CY060438**	**CY060439**	**CY060440**	**CY060441**	**CY060442**	**CY060443**
A/whooperswan/Mongolia/232/05	GQ907357	GQ907356	GQ907355	GQ907350	GQ907353	GQ907352	GQ907351	GQ907354

*Sequences submitted to Genbank are depicted in bold

#Submitted as draft sequence, due to low coverage, low quality, ambiguities, frameshifts, gaps, or other problems.

Table S2. Statistically supported migration rates between regions for the whole-genome dataset.

	PB2	PB1	PA	HA	NP	NA	M	NS
East to West	0.64[*†] (0.01:1.62)		**2.61 (0.29:5.35)**	**1.58 (0.10:3.47)**			1.43 (0.03:3.24)	**2.05 (0.44:4.16)**
East to Central	**0.93 (0.07:2.11)**	**0.87 (0.05:1.99)**	0.76 (0.04:1.74)	0.65 (0.00:1.55)	**1.48 (0.16:3.08)**			0.71 (0.02:1.71)
East to Oceania	**2.55 (0.67:4.81)**	**1.86 (0.40:3.67)**	**1.45 (0.24:3.01)**	**1.59 (0.19:3.27)**	**1.37 (0.16:3.08)**	0.89 (0.00:1.97)	1.15 (0.04:2.56)	
West to Central	**1.4 (0.25:2.81)**	**1.16 (0.13:2.40)**	**1.23 (0.18:2.54)**	**0.866 (0.12:1.84)**	**1 (0.01:2.21)**	**0.97 (0.78:1.98)**	**1.42 (0.16:2.91)**	**1.6 (0.37:3.11)**
West to East	**1.58 (0.35:3.12)**	**2.12 (0.39:4.27)**		**2.05 (0.47:3.96)**	2.13 (0.11:4.27)	**2.52 (0.56:4.69)**	**1.85 (0.24:3.83)**	**1.88 (0.39:3.70)**
West to Oceania						0.73 (0.00:1.66)		0.87 (0.02:1.90)
Central to West								
Central to East				0.81 (0.00:199)			1.06 (0.00:2.66)	
Central to Oceania								
Oceania to West				1.16 (0.02:2.35)		**0.95 (0.16:1.95)**		
Oceania to Central								
Oceania to East					0.6 (0.00:1.52)		0.92 (0.00:2.19)	

[*] Only migration rates with a BF higher than 8 are shown

[†] Migration rates with a BF higher than 100 are depicted in bold

Legends

Figure S1. Maximum likelihood trees of (a) PB2 and PB2, (b) PA and HA, (c) NP and NA and (d) MP and NS, displaying the genetic diversity of avian influenza A viruses in Eurasian wild birds. The taxa color indicates the bird species group from which the sample was isolated: Dabbling ducks – blue, diving and other ducks – pink, geese – orange, swans – green, fowl – black, gulls and other shorebirds – red.

Figure S2. Maximum likelihood trees of (a) PB2 and PB2, (b) PA and HA, (c) NP and NA and (d) MP and NS, displaying the genetic diversity in different locations in Eurasia. The taxa color indicates the regional location from where the sample was isolated: Oceania - cyan, East Eurasia - red, Central Eurasia - green, West Eurasia – blue.

Figure S3.

Maximum clade credibility (MCC) trees, summarizing the results of the Bayesian phylogenetic inference of (a) PB2 and PB2, (b) PA and HA, (c) NP and NA and (d) MP and NS, displaying the genetic diversity in different locations in Eurasia. The taxa color indicates the regional location from where the sample was isolated: Oceania - cyan, Central Eurasia - green, East Eurasia - red, West Eurasia - blue. The scale bar represents time in years

Figure S1a

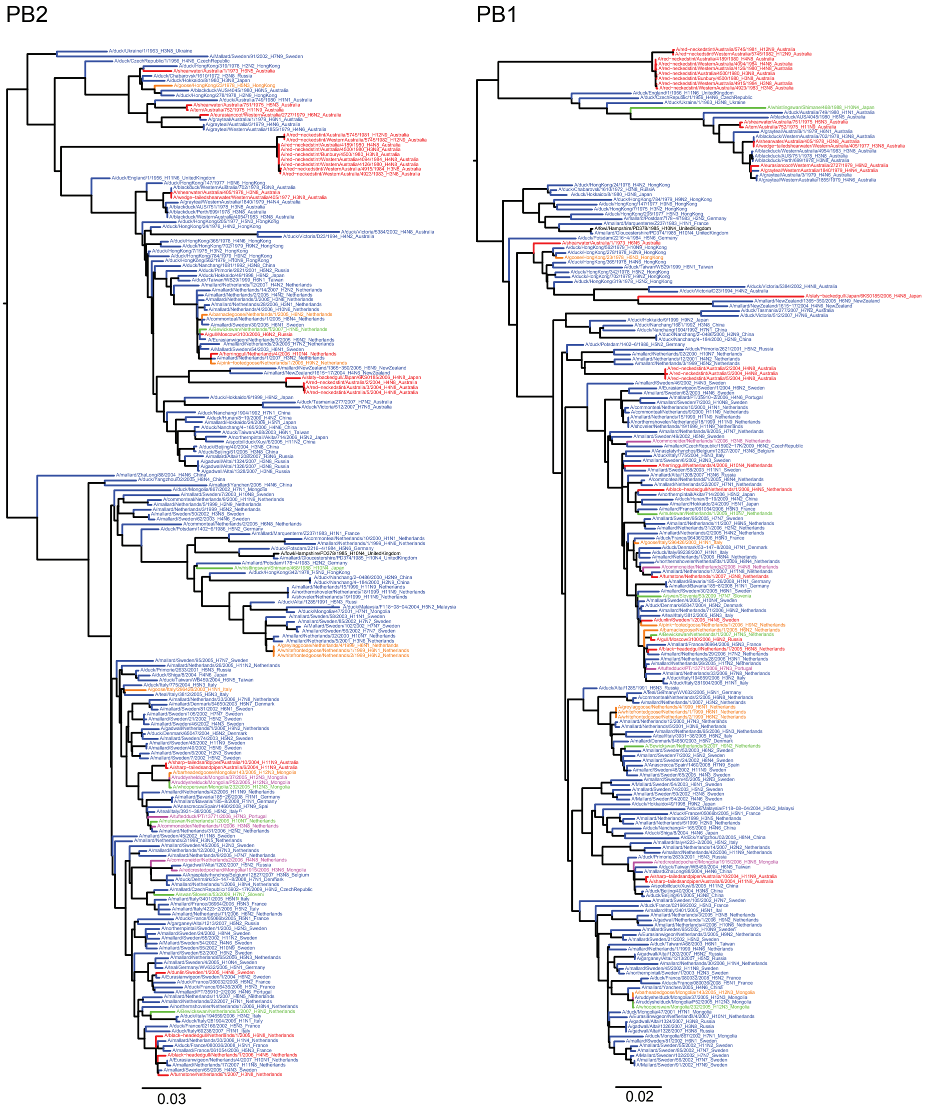

Figure S1b

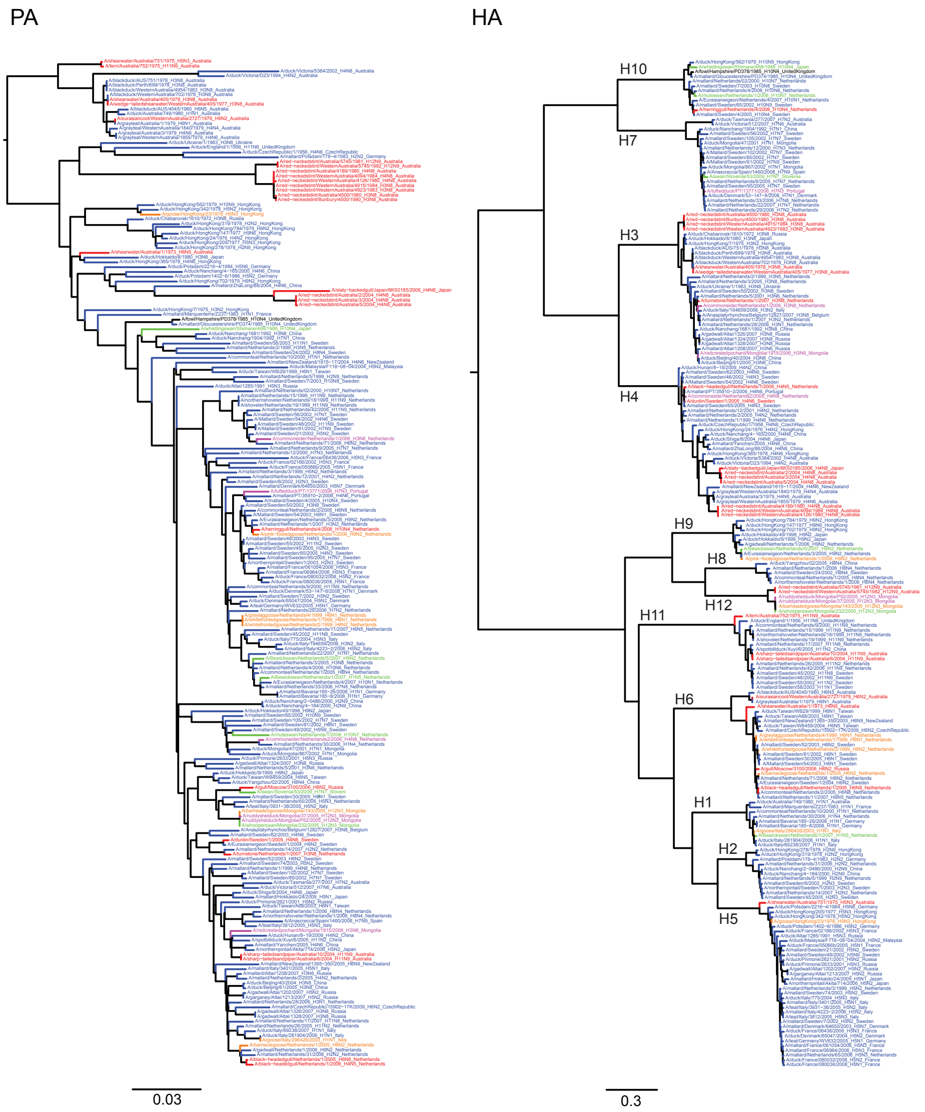

Figure S1c

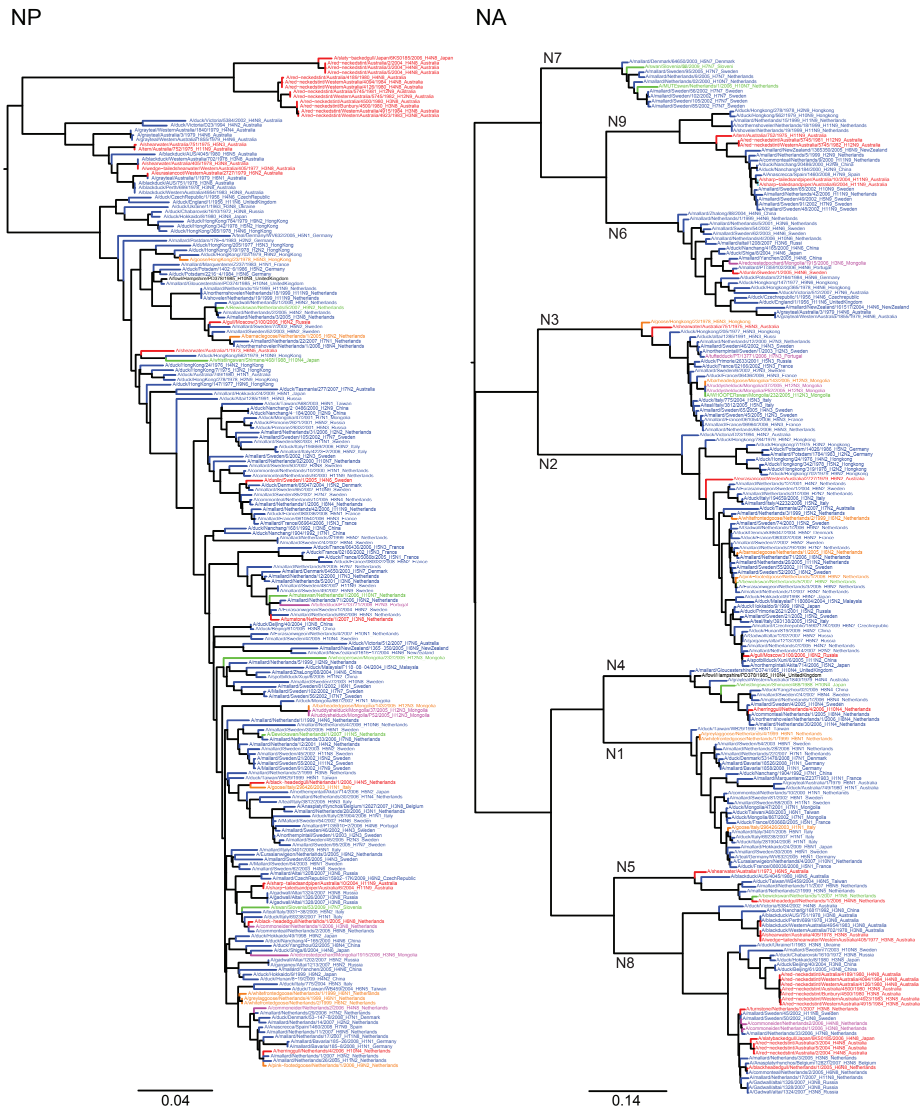

Figure S1d

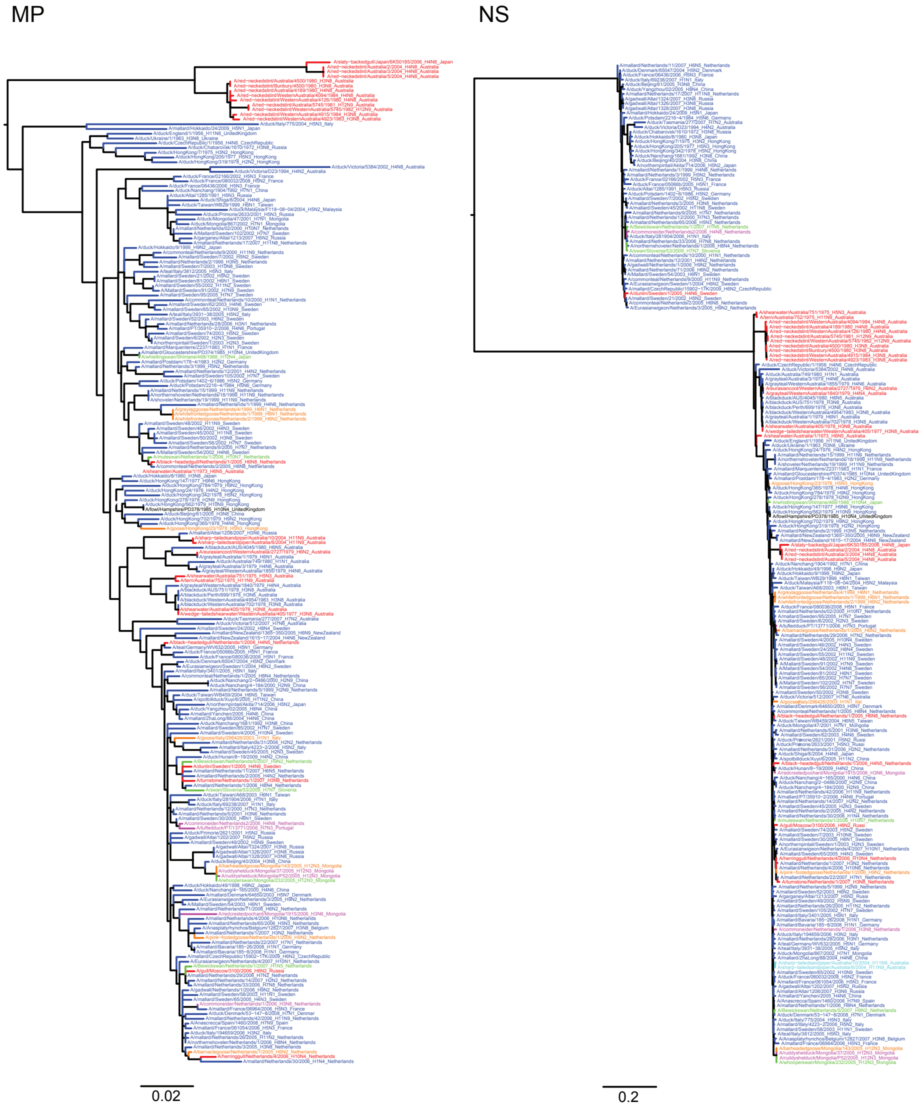

Figure S2a

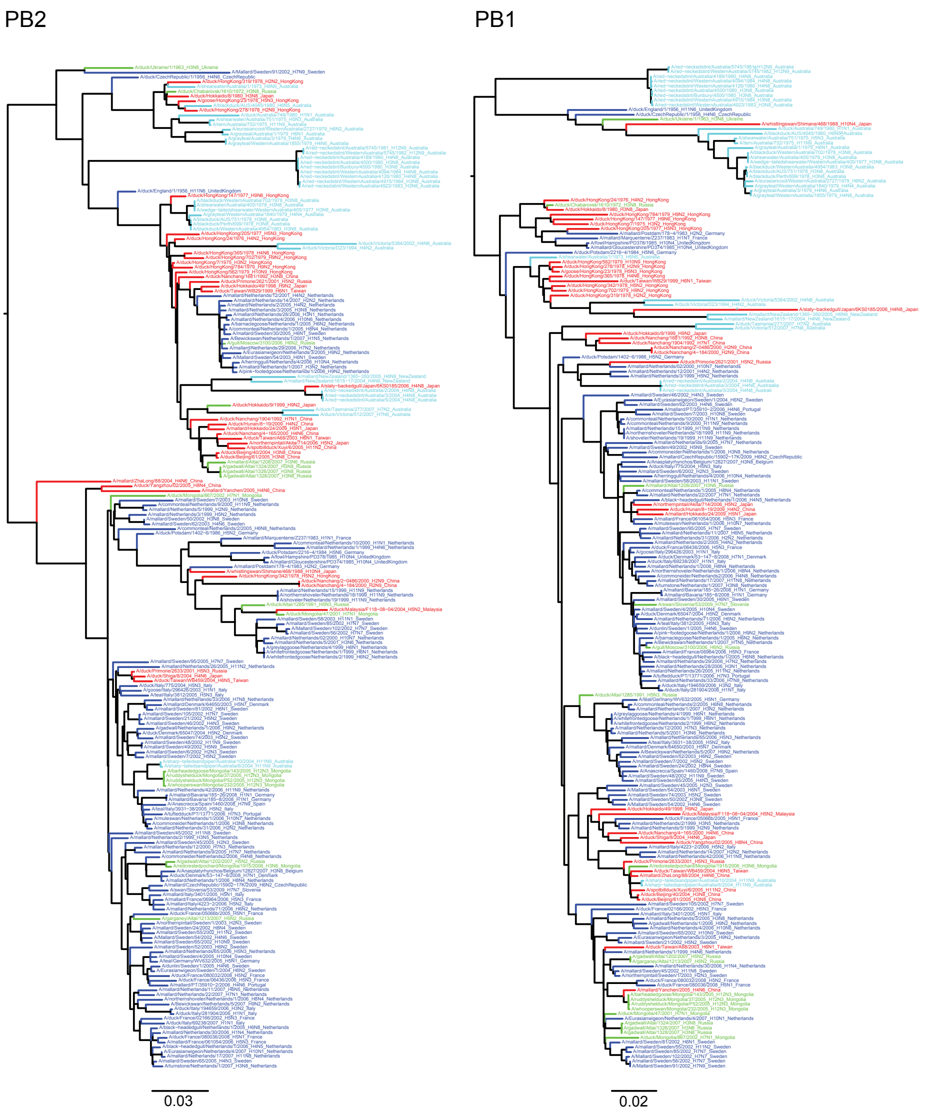

Figure S2b

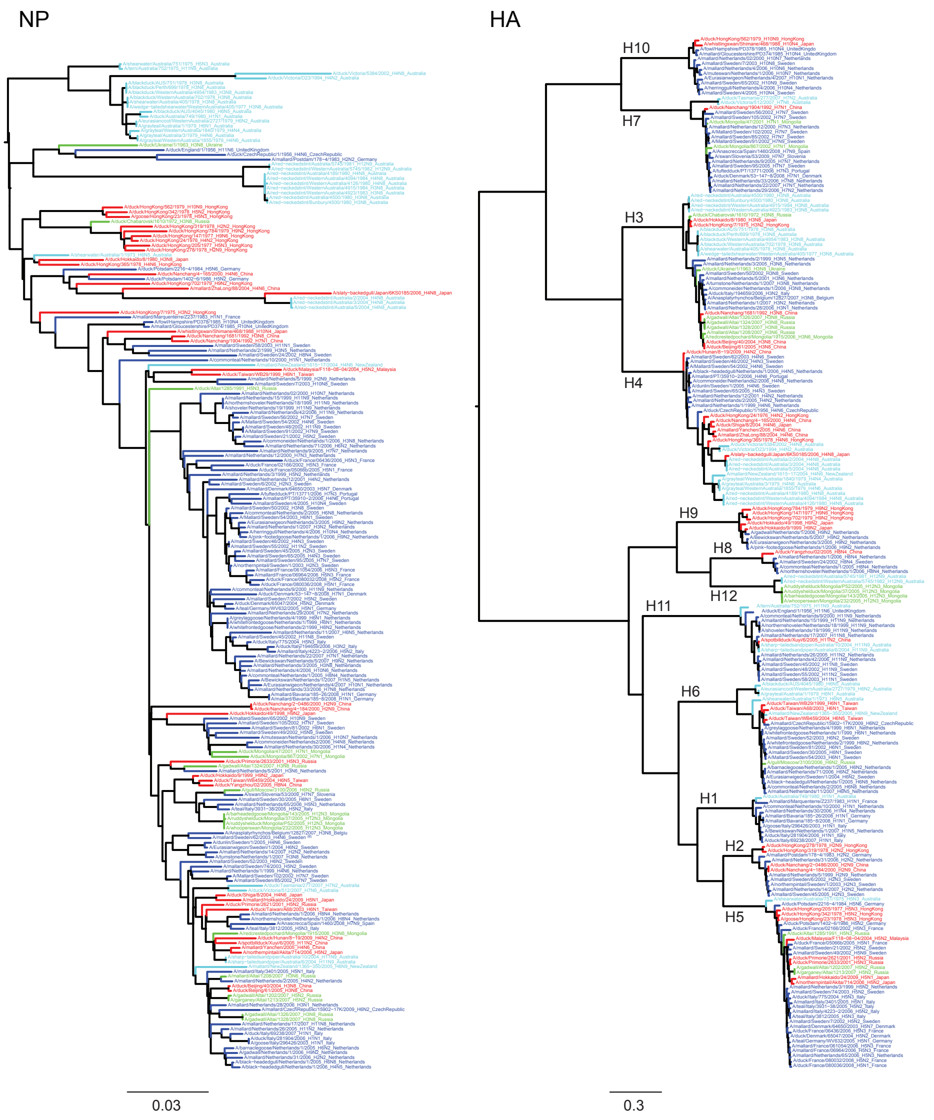

Figure S2c

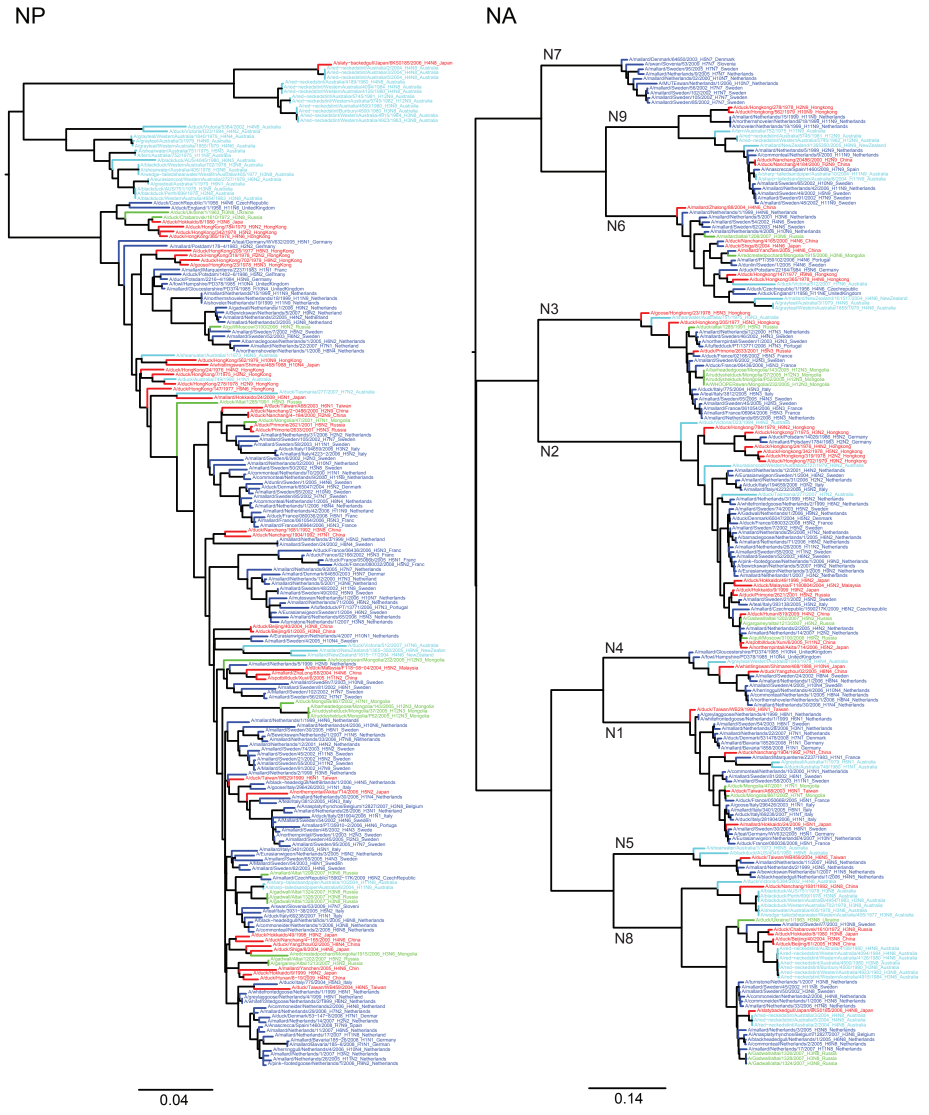

Figure S2d

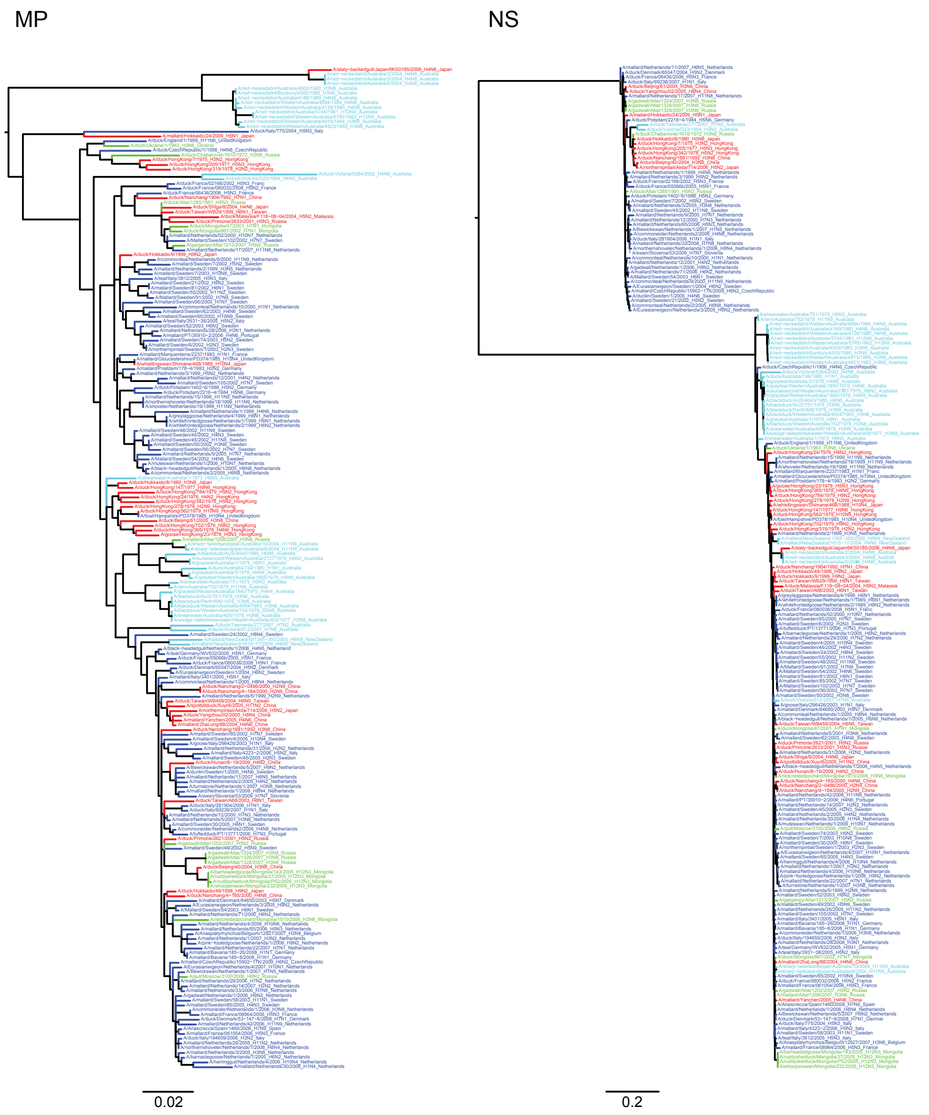

Figure S3a

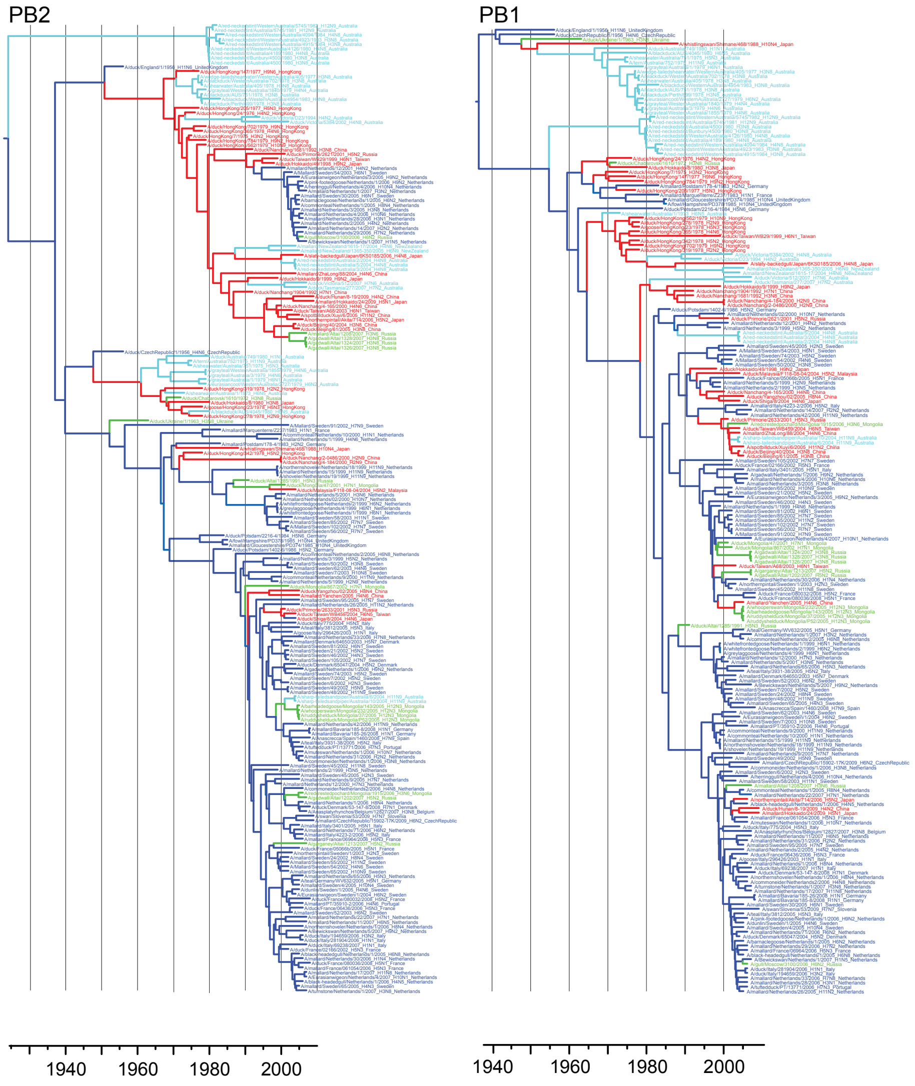

Figure S3b

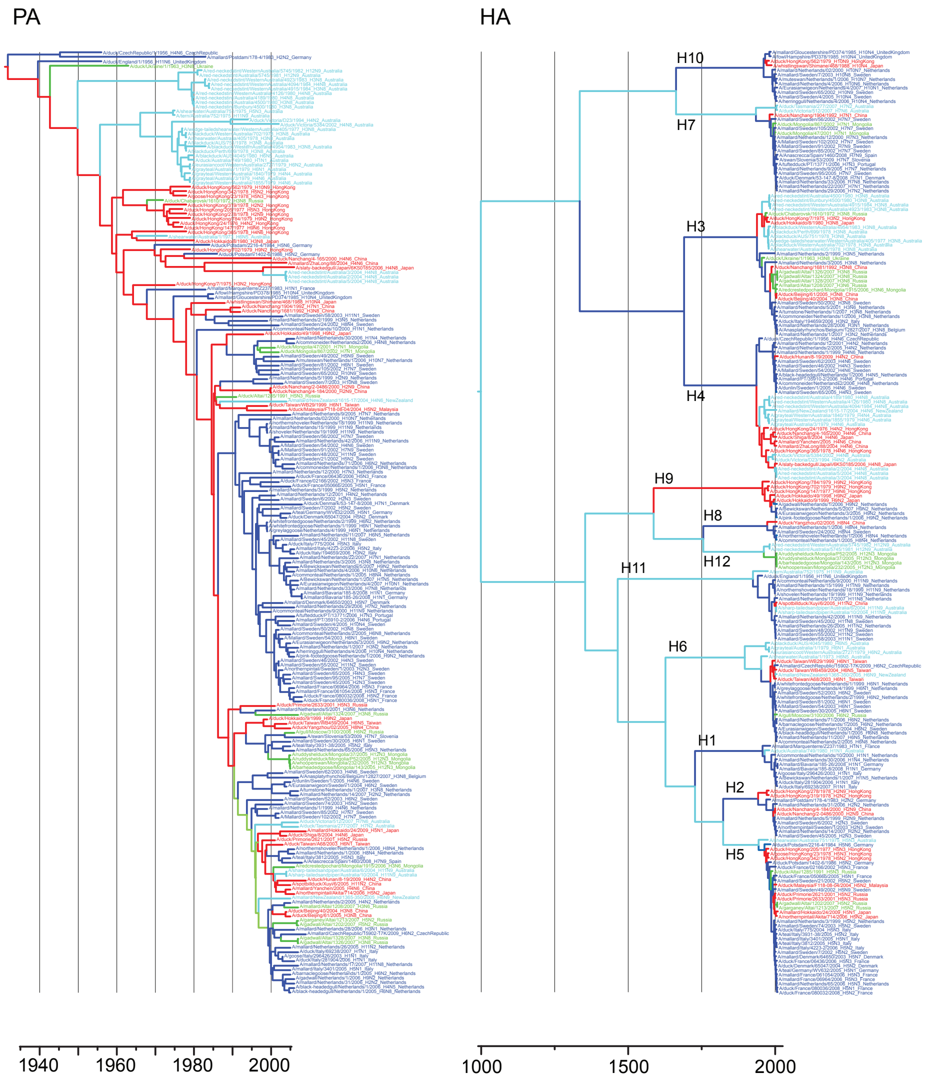

PA
HA
H10
H7
H3
H4
H9
H8
H12
H11
H6
H1
H2
H5
1940
1960
1980
2000
1000
1500
2000

Figure S3c

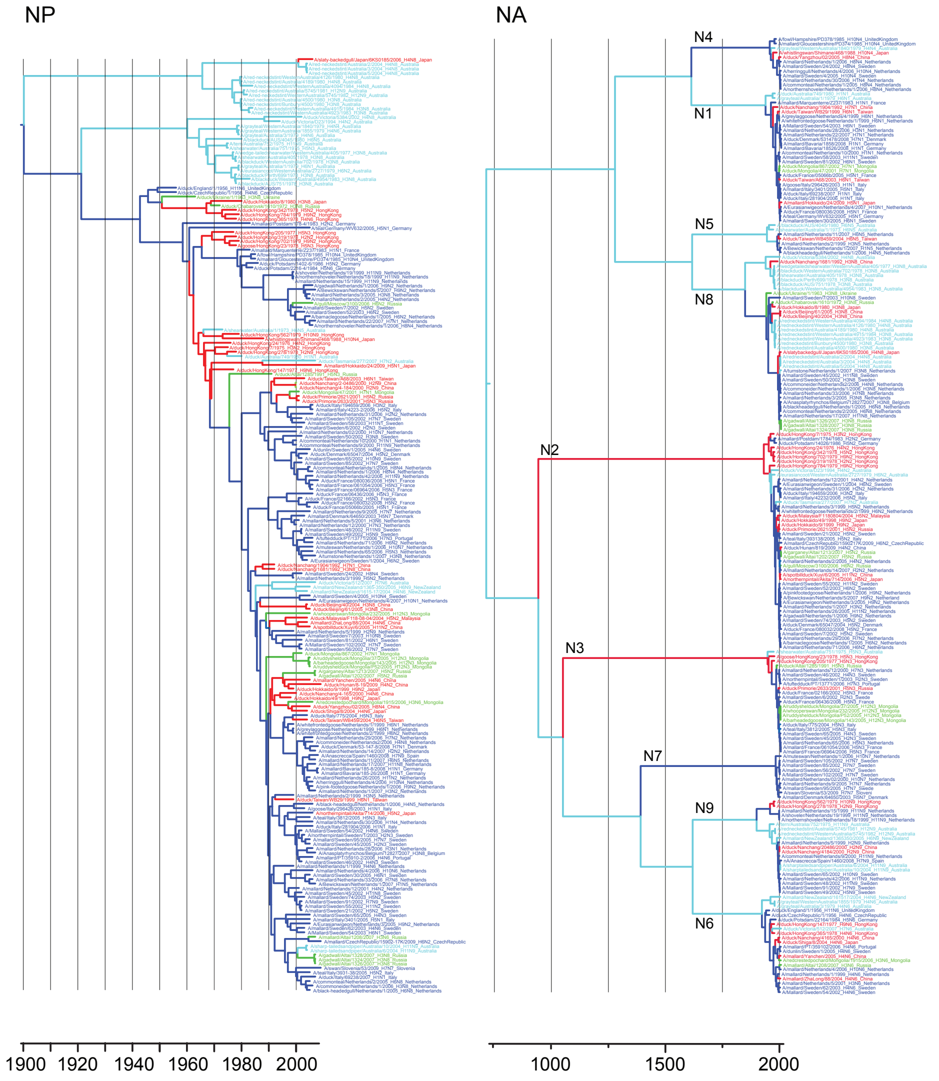

NP
NA
N4
N1
N5
N8
N2
N3
N7
N9
N6
1900
1920
1940
1960
1980
2000
1000
1500
2000

Figure S3d

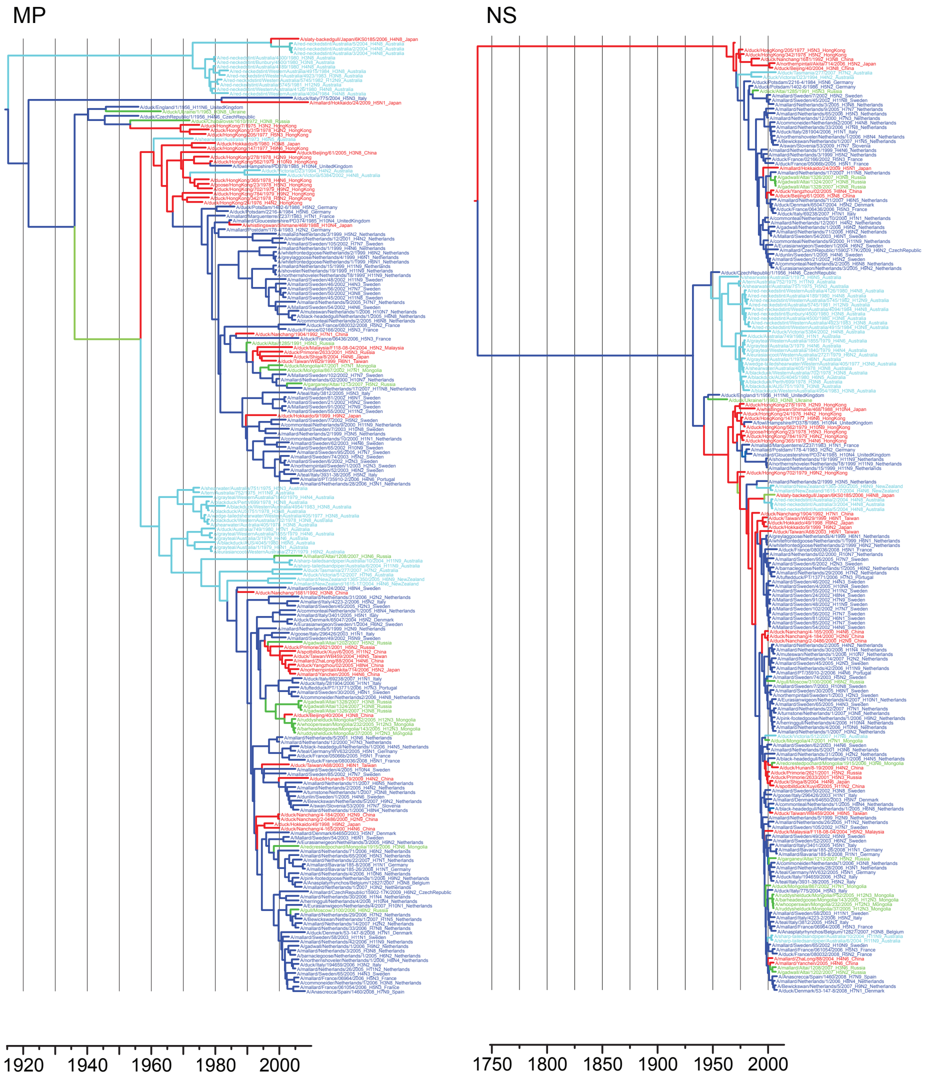

Supplement: Supplementary file 1 — Supplementary Data [file vir-96-08-2050-s001.pdf]
